# Supplementary material for: Safety, tolerability, and immunogenicity of SPVX02, a room temperature-stabilised tetanus-diphtheria vaccine, compared to two established tetanus-diphtheria booster vaccines: a multicentre, single-blind, randomised, first-in-human phase 1 trial in the UK
Source: eClinicalMedicine. 2026 Aug 5;99:104116. doi: 10.1016/j.eclinm.2026.104116 (PMC13616567; doi:10.1016/j.eclinm.2026.104116)
Supplement: Supplementary file 1 [file mmc1.pdf]

Supplementary file:

Safety, tolerability and immunogenicity of SPVX02, a room temperature-stabilised Tetanus-Diphtheria vaccine compared to Tetadif and diTeBooster: a multicentre, phase 1, blinded, randomised clinical trial.

Table of Contents

1. Neutralising Antibodies: Post-vaccination Geometric Mean Titres (GMTs) of Anti-Diphtheria 2
2. SPVX02 Drug Product - Stability Studies 3
   2.1 Results SPVX02 in-vivo potency testing 4
3. Immunogenicity Assessment – Antibody Responses to Tetanus and Diphtheria at baseline and Day 28................8
4. Certificate of Analysis SPVX02 Batch N° P126404A 9
5. Certificate of Analysis Tetadif Batch N° D0727-02 11
6. Certificate of Analysis diTeBooster Batch N° DT411A 12
7. SmPC Tetadif 15
8. SmPC diTeBooster 23
9. ELISA Method: Anti-Diphtheria Toxoid and Anti-Tetanus Toxoid 37
10. TNT Method: WHO – Annex 12 Diphtheria Antitoxin Assay in Vero Cell (in vitro toxin neutralisation) 38
11. Statistical Analysis Plan...45

List of Tables

1. Table S1. Neutralising Antibody Titres for Diphtheria.. 2
2. Table S2. . Stability Testing Panel and Analytical Methods for SPVX02 Drug Product...3
3. Table S3. In-Vivo Potency (Lethal Challenge Test) of Tetanus and Diphtheria Toxoids in SPVX02 (Lot Number P126402E) 6
4. Table S4. In-Vivo Potency (Lethal Challenge Test) of Tetanus and Diphtheria Toxoids in SPVX02 (Lot Number P126404A) 7

List of Figures

1. Figure S1. In-Vivo Potency of Tetanus and Diphtheria Toxoids in SPVX02 Engineering Batch (Lot Number P126402E). 5
2. Figure S2. Immunogenicity –Anti-Tetanus toxoid IgG titres at day 28 compared to day 0.. 8
3. Figure S3. Immunogenicity –Anti-Diptheria toxoid IgG titres at day 28 compared to day 0......................................8

1. Neutralising Antibodies: Post-vaccination Geometric Mean Titres (GMTs) of Anti-Diphtheria

Table S1 presents post-vaccination neutralising antibody responses against diphtheria at Day 28 following administration of a single dose of SPVX02, Tetadif or diTeBooster.

The table includes seroprotection rates and geometric mean titres (GMTs) with 95% confidence intervals.

For the analysis of geometric mean titres, the neutralising antibody titres were log10-transformed prior to analysis. Values above the upper limit of quantification (4.096 IU/mL) were set to the upper limit for calculation purposes. The proportion of participants with neutralising antibody titres ≥4.096 IU/mL was 50% for SPVX02, 45% for Tetadif and 85% for diTeBooster. Geometric mean titres (GMTs) were calculated by back-transformation of the mean log10 titres.

Table S1. Neutralising antibody titres for diphtheria

	Statistic	SPVX02 (N = 20)	Tetadif (N = 20)	diTeBooster (N = 20)
Seroprotection Rate	n	20	20	19
	(%)	(100.0)	(100.0)	(95.0)
	95% CI	83.16, 100.00	83.16, 100.00	75.13, 99.87
Longer-Term Seroprotection Rate	n	18	18	19
	(%)	(90.0)	(90.0)	(95.0)
	95% CI	68.30, 98.76	68.30, 98.76	75.13, 99.87
Geometric Mean Titres	n	20	20	20
	GMT	2.27	1.98	3.1
	95% CI	1.61, 3.21	1.38, 2.84	2.06, 4.68

Seroprotection is defined as IgG serum antibody titre of >= 0.1 IU/mL.
Longer-term seroprotection is defined as IgG serum antibody titre of >= 1.0 IU/mL.
95% CI = 95% Clopper-Pearson Confidence Interval.
GMT= Geometric mean titres.

2. SPVX02 Drug Product - Stability Studies

SPVX02 long-term stability study was initiated on 22nd December 2023. It aims to evaluate the stability of SPVX02 under controlled conditions of 30°C/75% Relative Humidity (RH) over a planned period of up to 4 years. This study is designed to demonstrate the maintenance of critical quality attributes and support the proposed shelf-life of the product.

The selected storage condition of 30°C/75% RH is aligned with ICH Q1A (R2) and WHO guidelines for stability testing in climatic zone IVb, representing hot and very humid environments. This condition reflects a worst-case scenario and supports the development of SPVX02 for use in regions with the most demanding environmental conditions. Demonstrating stability under these conditions (Climatic Zone IVb, 30°C/75% RH) is considered representative for global use, including less demanding regions, like Europe (climatic zone II, 25°C/60% RH).

The stability of SPVX02 is assessed through a comprehensive panel of stability tests to monitor all lyophilised and reconstituted SPVX02 vaccine attributes. The list of tests performed at each time point of the study are presented in the table below:

Table S2. Stability Testing Panel and Analytical Methods for SPVX02 Drug Product

Test	Method
Appearance	Visual inspection Ph. Eur. 0153
Reconstitution time	In-House
Reconstituted solution appearance	Visual check and Ph. Eur. 2.2.1 , 2.2.2
Extractable volume	Extractable volume Ph. Eur. 2.9.17
Water content	Karl Fisher method. Eur. 2.5.12 or Ph. Eur. 2.5.32
Osmolality	Ph. Eur. 2.2.35
Test for Endotoxins	Ph. Eur. 2.6.14
pH	Potentiometric determination of pH Ph. Eur. 2.2.3
Sterility	Direct inoculation. Ph. Eur. 2.6.1
Aluminium content	Aluminium in adsorbed vaccines Ph. Eur. 2.5.13
Sodium Chloride content	Potentiometric titration Ph. Eur 2.2.20
Identity of diphtheria	Immunodiffusion Ph. Eur. 2.7.1
Identity of tetanus	Immunodiffusion Ph. Eur. 2.7.1
CCI	USP <1207> / Ph. Eur. 3.2.9
Residual free detoxifying agent content	Formaldehyde content/ qualitative Ph. Eur.2.4.18
Degree of adsorption for tetanus	Flocculation test for toxoids Ph. Eur. 2.7.27
Degree of adsorption for diphtheria	Flocculation test for toxoids Ph. Eur. 2.7.27
Specific toxicity test for tetanus	Animal test in guinea pigs Ph.Eur. 0647
Specific toxicity test for diphtheria	Animal test in guinea pigs Ph.Eur. 0647
Potency test for tetanus	Challenge test in guinea pigs Ph. Eur. 2.7.8
Potency test for diphtheria	Challenge test in guinea pigs Ph. Eur. 2.7.6

The testing schedule for SPVX02 long-term stability study includes assessments at 0, 3, 6, 9, 12, 18, 24, 36 and 48 months. Additionally, the stability study includes an accelerated condition at 40°C/75% RH, with testing at 3 and 6 months and a freeze–thaw study, in which SPVX02 samples were subjected to three freeze–thaw cycles (-20°C and +40°C for 24 hours) to assess the impact of temperature fluctuations on product quality.

This stability study is ongoing and includes two batches of SPVX02: the engineering batch (Lot Number P126402E; stability study initiated on the 22nd December 2023) and the clinical trial batch (Lot Number P126404A; stability study initiated on the 30th September 2024). Both batches of SPVX02 were manufactured under GMP conditions and are subsequently analysed for 21 predefined quality attributes at each stability study timepoint. Testing timepoints include: 1 month at 40°C and 75% relative humidity (RH) with 3 freeze thaw cycles, 3, 6, 9, 12, 18, 24, 36 and 48 months at 30°C and 75% relative humidity, and 3 and 6 months at 40°C and 75% relative humidity.

This following section presents the in-vivo potency challenge study data generated to date, with data generated up to the 24 month timepoint for the SPVX02 engineering batch and up to the 12 month timepoint for the SPVX02 clinical batch.

In-vivo potency testing is performed according to the compendial method required by the European Pharmacopoeia, Ph. Eur. 2.7.6 for diphtheria and Ph. Eur. 2.7.8 for tetanus, for the assessment vaccine potency and is routinely used for batch release and stability evaluation of tetanus diphtheria vaccines.

2.1 Results SPVX02 In-vivo potency testing

Stability study with SPVX02 engineering batch (P126402E): Complete protection against supra-lethal doses of tetanus and diphtheria toxins was demonstrated in immunised guinea pigs across all tested conditions, including batch release, 1 month at 40°C/75% RH with three freeze–thaw cycles, long-term storage at 30°C/75% RH (up to 24 months), and accelerated conditions at 40°C/75% RH (up to 6 months) (see Figure S1 and Table S3).

Stability study with SPVX02 clinical batch (P126404A): Comparable potency was observed under the same conditions, with data available up to 12 months (see Table S4).

These findings indicate that reformulation of Tetadif bulk vaccine into SPVX02, as well as storage under elevated temperature conditions, does not adversely affect vaccine potency (see Table S3 and Table S4).

Figure S1: In-Vivo Potency of Tetanus and Diphtheria Toxoids in SPVX02 Engineering Batch (Lot Number P126402E).

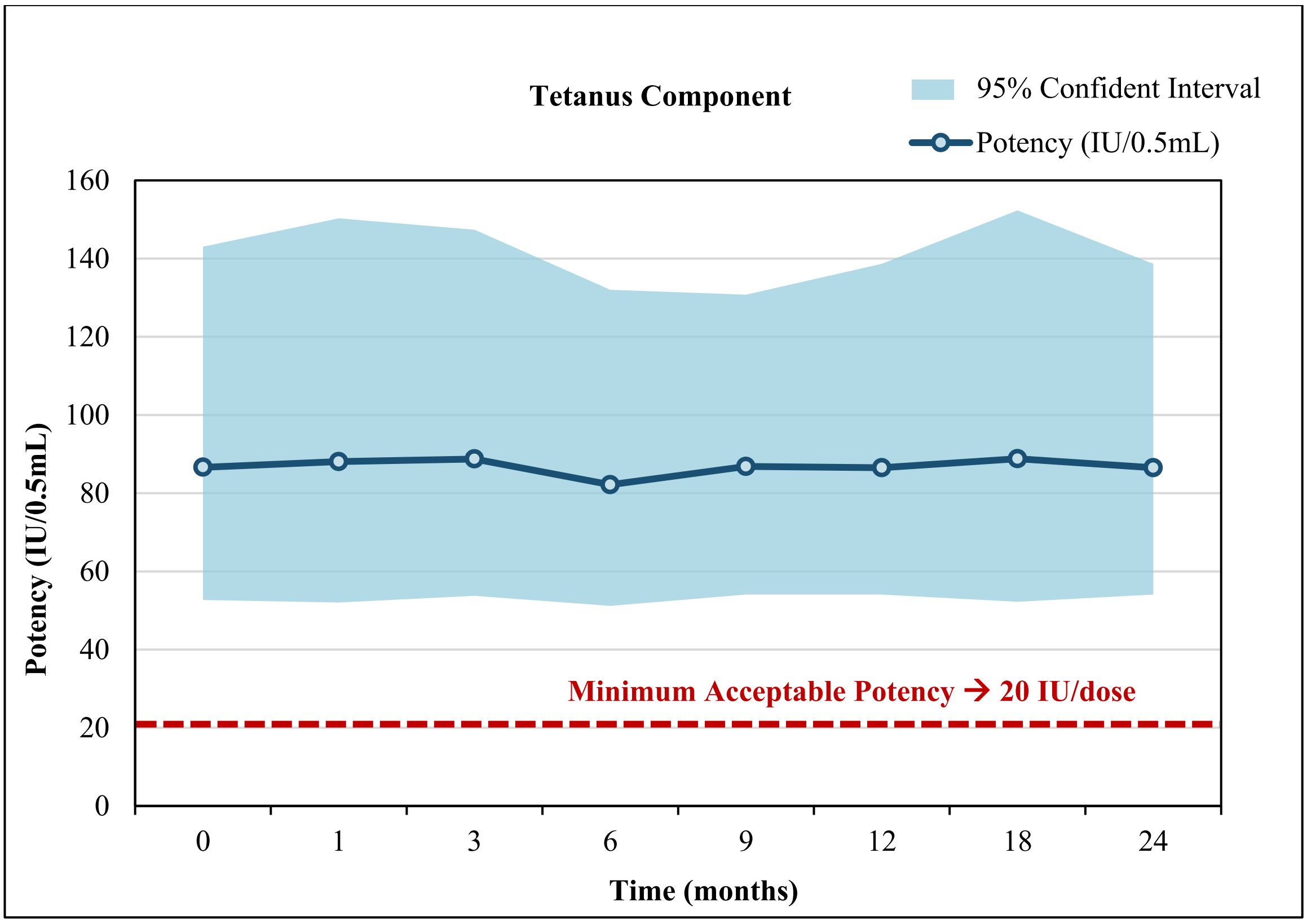

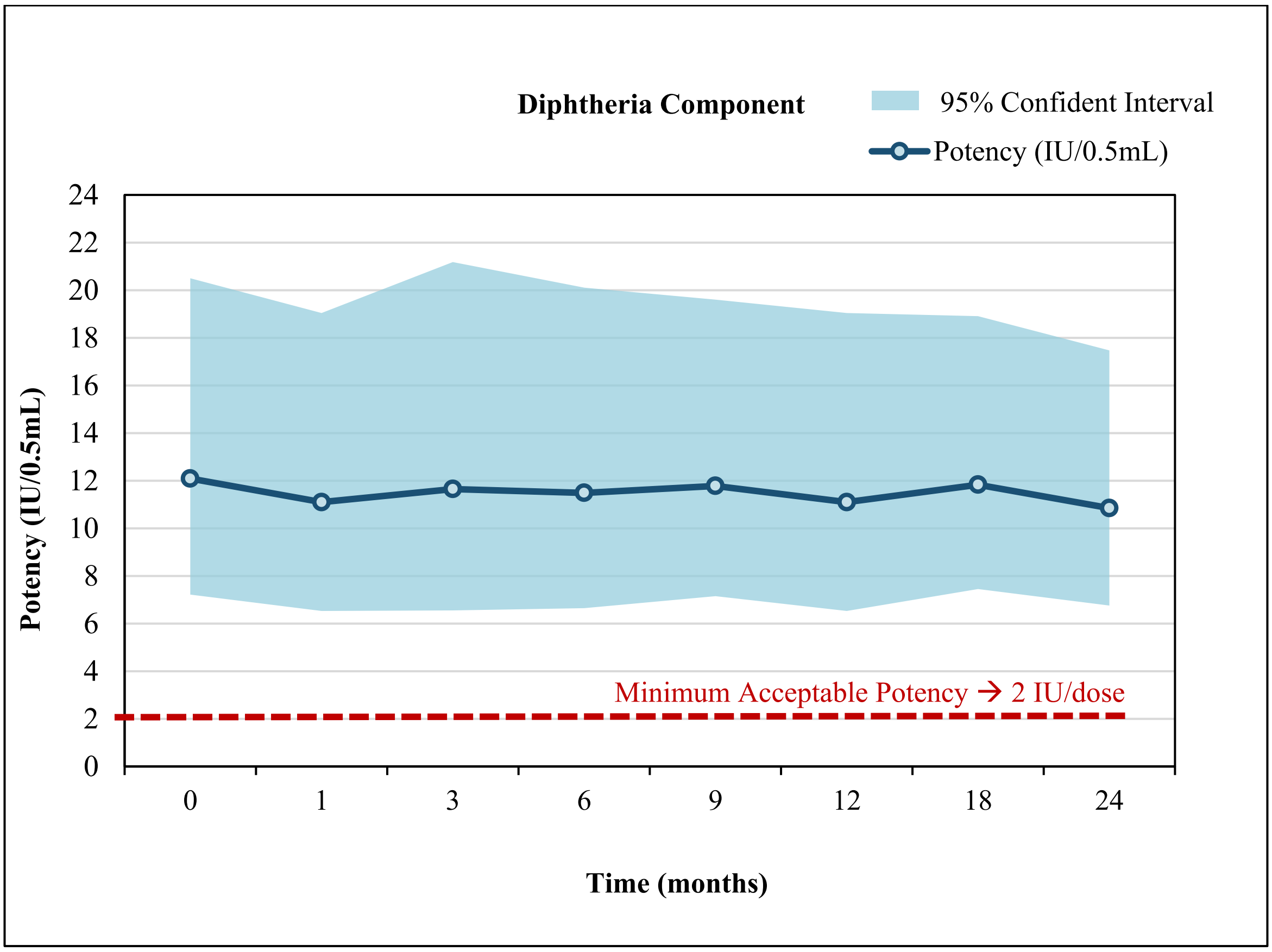

Potency values consistently remain above the minimum potency threshold of 20 IU/dose (Tetanus) and 2IU/dose (Diphtheria)

Table S3 In-Vivo Potency (Lethal Challenge Test) of Tetanus and Diphtheria Components in SPVX02 Drug Product (Lot Number P126402E)

TETANUS TOXIN CHALLENGE TEST										
	T=0 (batch release)	T=1 month[a] (freeze-thaw)	T=3 months at 30±2°C[b]	T=3 months at 40±2°C[c]	T=6 months at 30±2°C [b]	T=6 months at 40±2°C [c]	T=9 months at 30±2°C [b]	T=12 months at 30±2°C b	T=18 months at 30±2°C b	T=24 months at 30±2°C b
**Nº Units Admin**	**Nº Died/Nº Animals Challenged**									
16 IU	0/8	0/8	0/8	0/8	0/8	0/8	0/8	0/8	0/8	0/8
8 IU	3/8	3/8	2/8	1/8	2/8	2/8	2/8	2/8	3/8	2/8
4 IU	4/8	4/8	5/8	6/8	6/8	6/8	5/8	4/8	5/8	4/8
2 IU	8/8	7/8	7/8	7/8	7/8	7/8	8/8	8/8	7/8	8/8
	**Potency (95% CL) (IU/0.5 mL)**									
	**86.63** (52.67-143.05)	**88.07** (52.02-150.27)	**88.75** (53.76-147.37)	**89.80** (55.71-145.30)	**82.17** (51.17-132.03)	**82.17** (51.17-132.03)	**86.82** (54.05-130.77)	**86.50** (54.08-138.65)	**88.81** (52.22-152.34)	**86.50** (54.08-138.65)
**DIPHTHERIA TOXIN CHALLENGE TEST**										
	T=0 (batch release)	T=1 month[a] (freeze-thaw)	T=3 months at 30±2°C[b]	T=3 months at 40±2°C[c]	T=6 months at 30±2°C [b]	T=6 months at 40±2°C [c]	T=9 months at 30±2°C [b]	T=12 months at 30±2°C b	T=18 months at 30±2°C b	T=24 months at 30±2°C b
**Nº Units Admin**	**Nº Died/Nº Animals Challenged**									
40 IU	0/8	0/8	1/8	0/8	1/8	0/8	0/8	0/8	0/8	0/8
20 IU	3/8	3/8	2/8	2/8	2/8	3/8	2/8	3/8	1/8	2/8
10 IU	5/8	5/8	3/8	5/8	3/8	5/8	3/8	5/8	3/8	5/8
5 IU	7/8	7/8	7/8	7/8	8/8	7/8	8/8	7/8	8/8	8/8
	**Potency (95% CL) (IU/0.5 mL)**									
	**12.09** (7.22-20.50)	**11.10** (6.53-19.04)	**11.65** (6.55-21.18)	**11.09** (6.72-18.42)	**11.49** (6.65-20.11)	**11.10** (6.53-19.04)	**11.78** (7.15-19.60)	**11.10** (6.53-19.04)	**11.83** (7.45-18.91)	**10.85** (6.76-17.47)

[a] Data from freeze-thaw stability testing of SPVX02 stored at 21 days at 40±2°C / 75±5% RH then (-20±2°C for 24 hours: 40±2°C / 75±5% RH for 24 hours) x3

[b] Data from long-term stability testing of SPVX02 stored at 30±2°C 75±5% RH

[c] Data from accelerated stability testing of SPVX02 stored at 40±2°C 75±5% RH

Abbreviations: Admin=administered; CL=confidence limit; IU=international units; Nº=Number; RH=relative humidity; T=time.

Table S4 In-Vivo Potency of Tetanus and Diphtheria Components in SPVX02 Drug Product (Lot Number P126404A)

**TETANUS TOXIN CHALLENGE TEST**								
	T=0 (batch release)	T=1 month[a] (freeze-thaw)	T=3 months at 30±2°C[b]	T=3 months at 40±2°C[c]	T=6 months at 30±2°C [b]	T=6 months at 40±2°C [c]	T=9 months at 30±2°C [b]	T=12 months at 30±2°C [b]
**Nº Units Admin**	**Nº Died/Nº Animals Challenged**							
16 IU	0/8	0/8	0/8	0/8	0/8	0/8	0/8	0/8
8 IU	2/8	3/8	3/8	2/8	3/8	3/8	3/8	3/8
4 IU	4/8	4/8	4/8	5/8	5/8	5/8	4/8	4/8
2 IU	8/8	8/8	7/8	8/8	8/8	8/8	8/8	7/8
	**Potency (95% CL) (IU/0.5 mL)**							
	**86.50** (54.08-138.65)	**86.63** (52.67-143.05)	**88.07** (52.02-150.27)	**80.00** (50.41-126.97)	**86.82** (54.05-139.77)	**86.82** (54.05-139.77)	**86.63** (52.67-143.05)	**88.07** (52.02-150.27)
**DIPHTHERIA TOXIN CHALLENGE TEST**								
	T=0 (batch release)	T=1 month[a] (freeze-thaw)	T=3 months at 30±2°C[b]	T=3 months at 40±2°C[c]	T=6 months at 30±2°C [b]	T=6 months at 40±2°C [c]	T=9 months at 30±2°C [b]	T=12 months at 30±2°C [b]
**Nº Units Admin**	**Nº Died/Nº Animals Challenged**							
40 IU	0/8	0/8	0/8	0/8	0/8	0/8	0/8	1/8
20 IU	2/8	2/8	2/8	3/8	2/8	3/8	2/8	2/8
10 IU	5/8	4/8	4/8	4/8	4/8	4/8	3/8	4/8
5 IU	8/8	8/8	8/8	8/8	7/8	7/8	8/8	8/8
	**Potency (95% CL) (IU/0.5 mL)**							
	**11.76** (7.42-18.69)	**11.71** (7.40-18.58)	**11.76** (7.26-19.13)	**10.83** (6.58-17.88)	**11.97** (7.23-20.08)	**11.01** (6.50-18.78)	**10.81** (6.65-17.64)	**10.58** (6.18-18.19)

[a] Data from freeze-thaw stability testing of SPVX02 stored at 21 days at 40±2°C / 75±5% RH then (-20±2°C for 24 hours: 40±2°C / 75±5% RH for 24 hours) x3

[b] Data from long-term stability testing of SPVX02 stored at 30±2°C 75±5% RH

[c] Data from accelerated stability testing of SPVX02 stored at 40±2°C 75±5% RH

Abbreviations: CL=confidence limit; IU=international units; Nº=Number; RH=relative humidity; T=time.

3. Immunogenicity Assessment – Antibody Responses to Tetanus and Diphtheria at baseline and Day 28

Immunogenicity was evaluated by measuring anti-tetanus toxoid (anti-TT) and anti-diphtheria toxoid (anti-DT) IgG titres at baseline (Day 0) and 28 days post-vaccination (Day 28). As shown in Figures S2 and S3, all treatment groups (SPVX02, Tetadif, and diTeBooster) demonstrated a clear increase in antibody titres from baseline to Day 28.

Figure S2: Immunogenicity –Anti-Tetanus toxoid IgG titres at day 28 compared to day 0

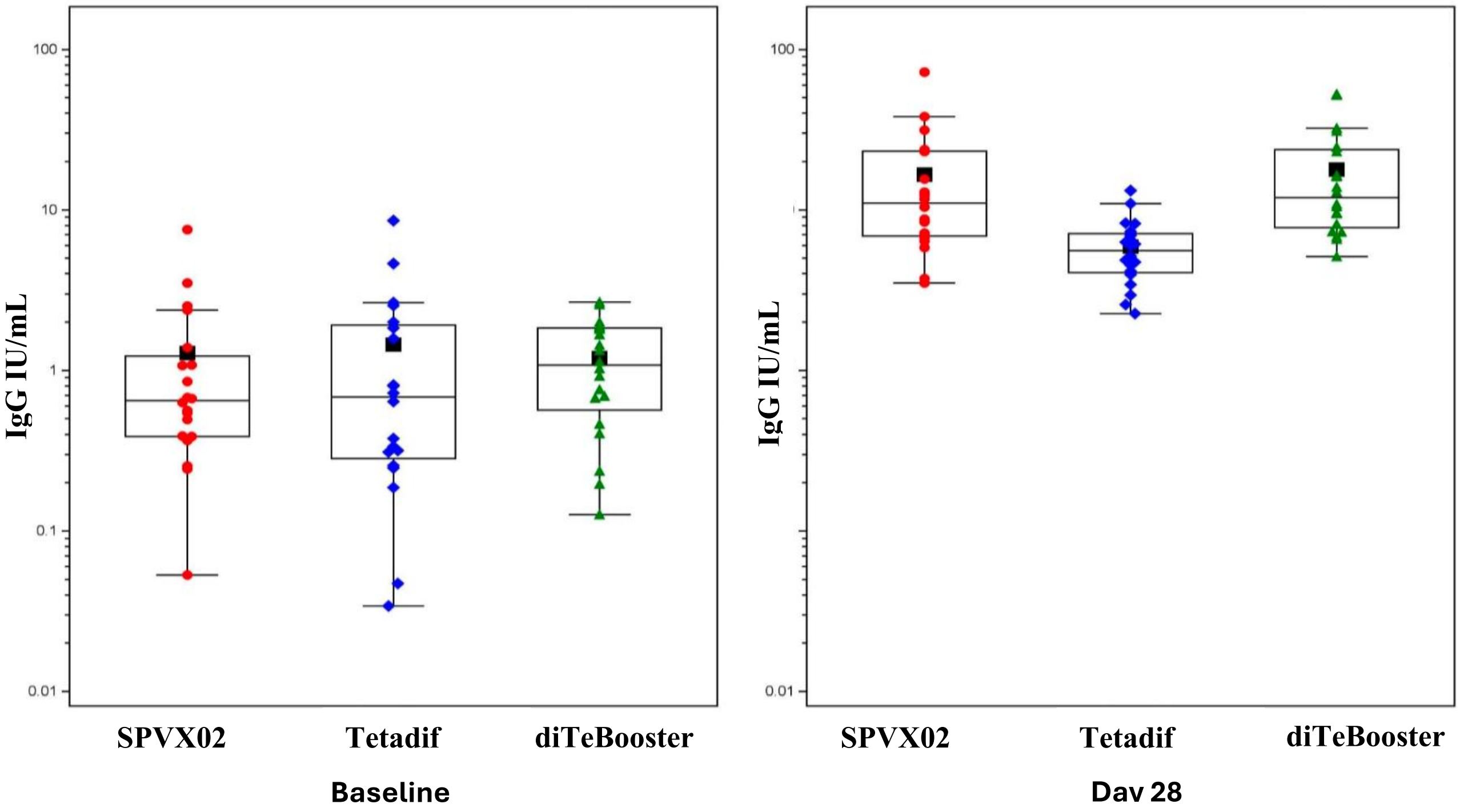

Figure S3: Immunogenicity –Anti-Diphtheria toxoid IgG titres at day at day 28 compared to day 0

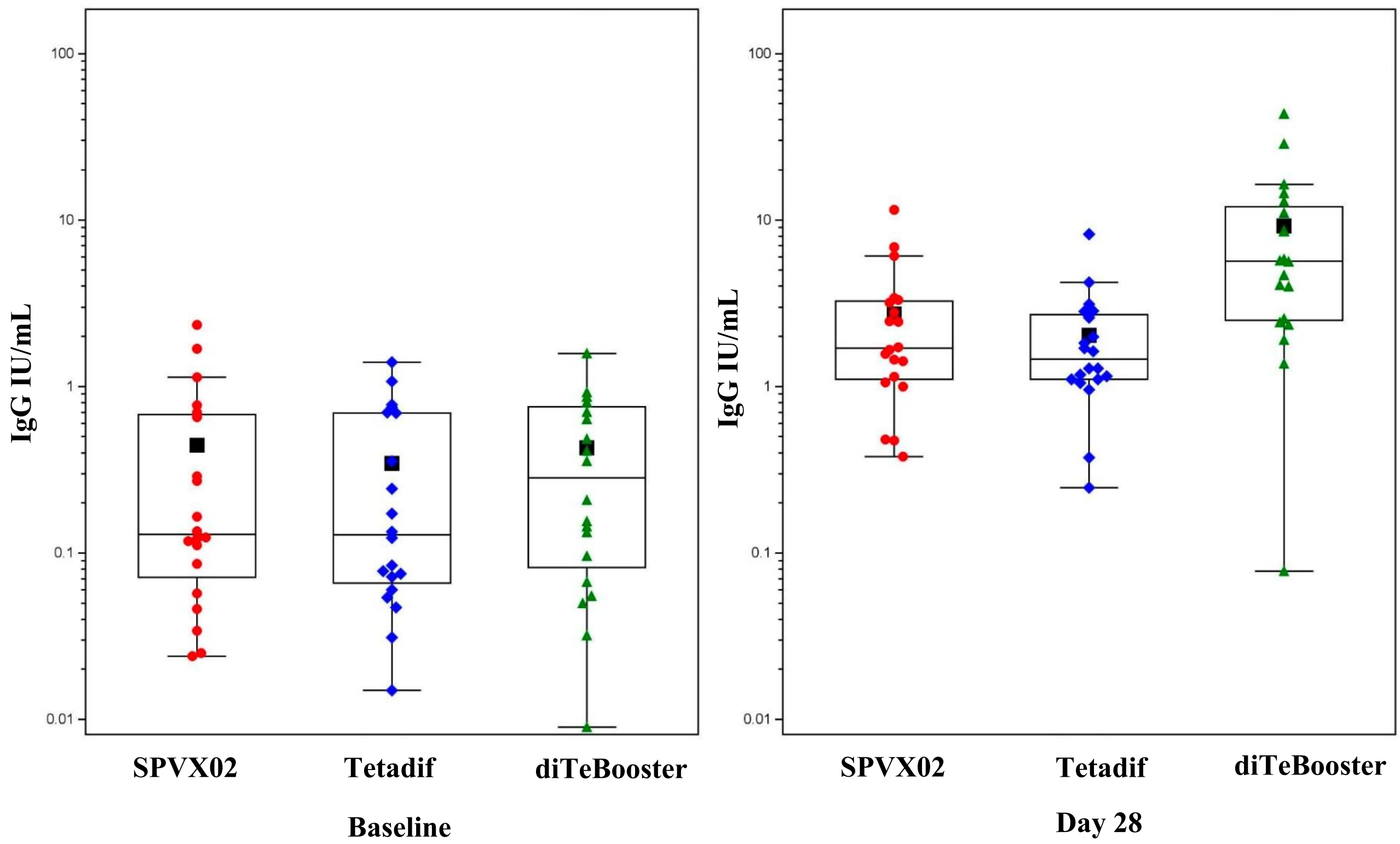

БУЛ БИО – НЦЗПБ ЕАД **BB – NCIPD EAD**

1504 София, бул. Янко Сакъзов 26
Факс: ** 359 2 943 34 55
Телефон Изпълнителен директор:
** 359 2 944 61 91
e-mail: bulbio@bulbio.com

1504 Sofia, 26 Yanko Sakazov blvd.
Fax: **359 2 943 34 55
Telephone Executive Director:
**359 2 944 61 91
E-mail: bulbio@bulbio.com

CERTIFICATE OF ANALYSIS

SPVX02 CTM

Tetanus-Diphtheria (adsorbed) (with reduced dose of diphtheria for adults)

Lot № P126404A

Date of manufacture: August 2024

№	Test/Method	Acceptance Criteria	Result
**1.**	Appearance	Suspension of white or grey particles dispersed in colourless or pale-yellow liquids and may form a sediment at the bottom of the container. Free of visible particles.	Pass
**2.**	pH	6.0 - 7.0	6.51
**3.**	Aluminium content	Maximum 1.25 mg/0.5 mL	0.60
**4.**	Sodium Chloride content	7.0 - 10.0 g/L	9.07
**5.**	Identity of diphtheria	Identity complies	Pass
**6.**	Identity of tetanus	Identity complies	Pass
**7.**	Sterility	Sterile	Pass
**8.**	Residual free formaldehyde	Maximum 0.2 g/L	Less than 0.2
**9.**	Degree of adsorption for tetanus	Not less than 80 %	100
**10.**	Degree of adsorption for diphtheria	Not less than 80 %	100
**11.**	Specific toxicity test for tetanus	None of the animals show abnormal reaction	Pass
**12.**	Specific toxicity test for diphtheria	None of the animals show abnormal reaction	Pass
**13.**	Potency test for tetanus component	Not less than 20 IU/0.5 mL	86.50 (54.08-138.65)
**14.**	Potency test for diphtheria component	Not less than 2 IU/0.5 mL	11.76 (7.42-18.69)

Conclusion:

SPVX02 CTM, Lot № P126404A has been successfully tested according to a list of standard qualified assays with defined acceptance limits (per Annex 1 of the Service Agreement between BB-NCIPD and Stablepharma, signed on 13th of June 2023).

Date of issue: 04.12.2024

Head of Quality Control Department:

R. Alexiev, PhD

Confidential level 1

CERTIFICATE OF ANALYSIS

Certificate N°: **0280/2024** Batch number: **P126404A**

Product name: **SPVX02 CTM** Code Patheon: **364101**

Client: **Stablepharma Ltd.**

Package size and type: **Bulk**

Results

Test description	Acceptance Criteria	Results
Moisture Content (Karl Fisher Method)	≤ 3.0%	0.9%
Appearance (LYO)	Stoppered and crimped glass vial containing a white lyoplug of approximately 5 mm height without signs of collapse	Stoppered and crimped glass vial containing a white lyoplug of approximately 5 mm height without signs of collapse
Appearance (Rec.Sol)	Suspension of white or grey particles dispersed in a colourless or pale yellow liquid and may form a sediment at the bottom of the container	Suspension of white particles dispersed in a colourless liquid and may form a sediment at the bottom of the container
pH (Rec.Sol)	6.0 – 7.0	6.5
Extractable volume	Not less than 0.5 ml (1 dose)	0.5 ml
Osmolality	500-750 mOsm/Kg	687 mOsm/Kg
Reconstitution time	<1 minute with gentle mixing – no visible residue as an undissolved matter	21 seconds – no visible residue as an undissolved matter
Sterility (direct inoculation)	Sterile	I° test: Sterile<br>II° test: Sterile
Bacterial Endotoxins	≤ 20 EU/mL	< 5 EU/mL

The material identified above has been analyzed and found to be COMPLIANT with release specification SPFS0692.

Printed on

Monza, 14/11/2024

Quality Control Manager

Electronically signed by: Riccardo Taribello Reason: Approver of the GxP document Date: Nov 14, 2024 14:10 GMT+1

Patheon Italia S.p.A. Societa' con Unico Azionista, Soggetta a direzione e coordinamento di "Patheon Inc." - Capitale Sociale EUR 104.000,00 i.v. - Codice fiscale e iscrizione al Registro delle Imprese di Monza e Brianza n. 01924610346 REA: MB-1566638 Partita IVA: IT 12584850155 - Sede Legale: Viale G.B. Stucchi 110 20900 Monza MB – Italia Italia – Sede Secondaria: Ferentino: 2° Trav. SX, Via Morolense n° 5, 03013 Ferentino (FR), Italia.

БУЛ БИО - НЦЗПБ ЕООД **BB - NCIPD Ltd.**

1504 София, бул. Янко Сакъзов 26
Факс: **359 2 943 34 55
Телефон Управител:
**359 2 944 61 91
e-mail: bulbio@bulbio.com

1504 Sofia, 26 Yanko Sakazov blvd.
Fax: **359 2 943 34 55
Telephone General Manager:
**359 2 944 61 91
e-mail: bulbio@bulbio.com

CERTIFICATE of ANALYSIS

TETADIF

Tetanus - Diphtheria toxoid (adsorbed) (with reduced dose of diphtheria for adults)

Susp. inj., amp – 0.5 ml - 1 dose, IM

Lot №: D0727-02

Date of manufacture: April 2023

№	Test / Method	Acceptance Criteria	Result
**1.**	Appearance	Suspension of white or gray particles dispersed in colorless or pale yellow liquids and may form a sediment at the bottom of the container	Pass
**2.**	Identity	Identical	Pass
**3.**	pH	6.0 – 7.0	6.38
**4.**	Adjuvant	Aluminium hydroxide<br>not more than 1.25 mg / 0,5ml	0.59 mg / 0.5 ml
**5.**	Sodium chloride	7.0 – 10.0 g / L	8.63 g / L
**6.**	Free formaldehyde	Not more than 0.2 g / L	< 0.2 g / L
**7.**	Sterility	Sterile	Sterile
**8.**	Degree of adsorption*	Not less than 80 per cent	100 per cent
**9.**	Specific toxicity - in guinea-pigs*	None of the animals shows abnormal reaction	Pass
**10.**	Potency*<br>- tetanus component<br><br>-diphtheria component	Lower confidence limit (P=0.95) of the estimated potency<br>Not less than 20.0 IU/dose<br><br>Not less than 2.0 IU/dose	82.22 IU / 0,5 ml<br>(50.06-135.23)<br><br>10.74 IU / 0,5 ml<br>(6.08-19.16)
**11.**	Extractable volume	Not less than 0.5 ml	Pass
**12.**	Storage condition	Store at 2°C - 8°C; Do not freeze	
**13.**	Expiry date	36 months of the start date of the last satisfactory potency determination (i.e. the date on which the test animals were immunized with the vaccine) is taken as the start date for the shelf-life	March 2026

* - Test is performed at stage final bulk

Conclusion:

Tetadif Lot № D0727-02 amp – 0.5 ml – 1 dose meets all national requirements and satisfies Part A of the WHO Recommendations to assure the quality, safety and efficacy of DT based vaccines (WHO TRS No.980, 2014) and European Pharmacopoeia.

The Vaccine has been produced in a firm with approved Quality Management System Standards: ISO 9001:2015 and ISO 14001:2016.

Date of issue: 27.06.2023

Head of Quality Control Department:
V. Milanova, PhD

Confidentiality Level 1

Certificate of Analysis (CoA)

diTeBooster

Batch DT411A

Batch no.: DT411A
Expiry date: 05-2026
Volume of bulk: 10565.4 mL
Volume of final bulk: 60.0 L

Used components:
Diphtheria toxoid AJV bulk, batch no.: D 94
Tetanus toxoid AJV bulk, batch no.: T 332

**Test**	**Results**	**Requirements**
**BULK (B)**		
B 1 Lf- concentration	Di: 13.1 Lf/mL<br>Te: 12.6 Lf/mL	Di: 12.5 ± 2.5 Lf/mL final bulk<br>Te: 12.5 ± 2.5 Lf/mL final bulk
**FINAL BULK (FB)**		
FB 1 Sterility	No growth	No growth
**FINAL LOT (FL)**		
Before labelling (FL-)		
FL 1 Al-concentration	First, day 1: 0.9 mg Al/mL<br>Last, day 1: 1.0 mg Al/mL	0.9-1.1 mg Al/mL
FL 2 Sterility	No growth	No growth
FL 3 Pyrogens	Complies<br>(0.10°C/3 rabbits)	Complies<br>(≤ 1.15°C/3 rabbits)<br>(≤ 2.80°C/6 rabbits)
FL 4 pH	7.2	6.5 ≤ pH ≤ 7.5
FL 5<br>Di:Potency lower confidens limit	Di: 21 IU/0.5mL	Di: $P=0.95_{\text{lower c.l.}}$ ≥2 IU/0.5mL
Te Potency	Te: ≥ 20 IU/0.5mL	Te: ≥ 20 IU/0.5mL

After labelling (FL+)

FL 6 Volume	0.58 mL/syringe	0.56-0.61 mL/syringe
FL 7 Appearance	Suspension of white particles in colourless liquid	Suspension of white or grey particles in colourless or light yellow liquid
FL 8 Identification	Di: Positive in eluate<br>Te: Positive in eluate<br>aP: Negative in eluate	Di and Te: Positive in eluate<br><br>aP: Negative in eluate
FL 9 Degree of adsorption	Di: 100% (0 Lf/mL in supernatant)<br>Te: 100% (0 Lf/mL in supernatant)	Di: 100% (0 Lf/mL in supernatant)<br>Te: 100% (0 Lf/mL in supernatant)

Date of release: 18.04.2024

18-04-2024

X Kim Skjold Rehmeier

Signed by: Kim Skjold Rehmeier (KIRE)

Delegated Qualified Person
MSc Pharmacy
QA Operations Department
Quality Assurance Area

SUMMARY OF PRODUCT CHARACTERISTICS

1. NAME OF THE MEDICINAL PRODUCT

TETADIF suspension for injection
Diphtheria and tetanus vaccine (adsorbed, reduced antigen(s) content)

2. QUALITATIVE AND QUANTITATIVE COMPOSITION

TETADIF is a suspension of purified and adsorbed tetanus and diphtheria toxoid onto aluminum adsorbent. They are produced from toxins of *Corynebacterium diphtheriae* and *Clostridium tetani* by detoxification and purification. Thiomersal is used as a preservative in the manufacturing of multi-dose vials. No preservative are used in the manufacturing of single-dose product (ampoules).

One dose (0.5 ml) contains:

Purified tetanus toxoid*	not less than 40 International Units (IU) (10.0 Lf)
Purified diphtheria toxoid*	not less than 4 International Units (IU) (3.0 Lf)

*Adsorbed onto aluminium hydroxide (Al^{+++}) not more than 1.25 mg

The vaccine in multi-dose vials contains the preservative thiomersal – not less than 0.05 mg.
See section 4.3.

Excipient with known effect
The vaccine contains less than 1 mmol sodium (23 mg) per dose, i.e. it is essentially 'sodium-free'.

For the full list of excipients, see section 6.1.

3. PHARMACEUTICAL FORM

Suspension for injection.

TETADIF is a suspension of white or grey particles dispersed in colourless or pale yellow liquids.

4. CLINICAL PARTICULARS

4.1 Therapeutic indications

TETADIF is indicated for:
1. Re-immunization against tetanus and diphtheria of children over 7 years of age and adults.
2. Primary immunization against tetanus and diphtheria started after 7 years of age.
3. Immunization after injury or burn with a risk of tetanus infection, if re-immunization against diphtheria in necessary.

4.2 Posology and method of administration

Posology
The single immunization dose of TETADIF is 0.5 ml.
The vaccination schedule shell be in accordance with the National or Referral Immunization Calendar.

Primary immunization
Primary immunization against tetanus and diphtheria, started after 7 years of age is performed three times, administered deep INTRAMUSCULARLY into the deltoid muscle of the arm: the first two doses of 0.5 ml at intervals of not less than 30 days between each dose and the third dose of 0.5 ml -

from 6 to 12 months after the second dose. In case of missed a dose, it should be administered whenever is possible.

Re-immunization
Subsequent re-immunizations have to comply with a re-immunization schedule according to the National or Referral Immunization Calendar.

Vaccination following injuries or burns with a risk of tetanus infection, if re-immunization against diphtheria is necessary, is performed by administering 0.5 ml INTRAMUSCLARLY, deep into the deltoid muscle of the arm.

Paediatric population
The safety and efficacy of TETADIF in children below 7 years of age have not been established.

Method of administration
The vaccine is administered deep INTRAMUSCLARLY (i.m.) into the deltoid muscle of the arm. The ampoule/ vial must be shaken prior to injection until a homogenous whitish suspension is obtained.
The vaccine is drawn from the ampoule (vial) using a sterile syringe and needle. After the air bubbles have been expelled from the syringe, the needle is replaced with a new dry sterile needle which is used for the injection.

4.3 Contraindications

Hypersensitivity to the active substance(s) or to any of the excipients of the vaccine listed in section 6.1.

The drug product contains thiomersal, as a preservative in multi-dose vials and is possible to cause an allergic reaction to you/ your child.

Anamnestic data for hypersensitivity reactions to previous vaccine administration.
General contraindications for immunizations:
1. Acute infectious diseases, convalescence period included.
2. Fever conditions.
3. Active tuberculosis.
4. Decompensated heart failure.
5. Diabetes mellitus, thyrotoxicosis, decompensated adrenal insufficiency.
6. Acute central nervous system inflammations – meningites, encephalites, meningoencephlalites.
7. Chronic active hepatites and biliary cirrhosis.
8. Infections of the urinary tract.
9. Nephrotic syndrome.
10. Autoimmune diseases.
11. Allergy, including medical data for shock, Oedema Quincke and other severe allergic reactions to allergens present in vaccines.
12. Epilepsy.

At the presence of contraindications the physician assesses the risk of TETADIF administration or tetanus or diphtheria disease.

4.4 Special warnings and precautions for use

Vaccine administration needs to be preceded by taking the patient’s medical history and performing a medical examination.
HIV infected patients are immunized, resp. re-immunized with TETADIF following the standard immunization schedules.
The effect of immunization is possible to be reduced in immunodeficiencies or in patients subjected to immunosuppressive treatment.

In such cases, it is recommended to postpone the immunization (if scheduled) until the immunosuppressive treatment is withdrawn.
Caution should be exercised in patients with coagulation disorders. The vaccinated person should remain under medical supervision for 30 minutes after immunization due to the possibility of an anaphylactic reaction.

Traceability
In order to improve the traceability of biological medicinal products, the name and the batch number of the administered product should be clearly recorded.

4.5 Interaction with other medicinal products and other forms of interaction

Immunization (resp. re-immunization) with TETADIF may be administered concomitantly with other vaccines. TETADIF is compatible with poliomyelitis, hepatitis B, measles, rubella, mumps, rabies, flu and yellow fever vaccines. The products listed above need to be administered using a different needle and syringe and at a different injection site than TETADIF.
The vaccine could be administered either concomitantly or separately in patients with injected immunoglobulins. The immunoglobulin injection site needs to be different from the one used for TETADIF administration.

There are no data available for interaction of the vaccine with other medicinal products.

4.6 Fertility, pregnancy and lactation

Pregnancy
No studies have been done to investigate the reproductive toxicity of the vaccine. However, immunization during pregnancy is not recommended.

Breastfeeding
There are no data indicating administration of the vaccine during the lactation.

Fertility
TETADIF has not been evaluated in fertility studies.

4.7 Effects on ability to drive and use machines

There are no studies indicating that TETADIF may impair the attention and the ability to drive and use machines. TETADIF has no or negligible influence on the ability to drive and use machines.

4.8 Undesirable effects

The adverse reactions after administration of TETADIF are:
Frequency is not known (cannot be estimated from the available data), according MedDRA-system organ class database).
General disorders and administration site conditions:
Local reactions may occur at the vaccine injection site, for example: Pain, redness, mild swelling and induration that may resolve in 1 - 2 days.
In some cases, there may be low-grade fever that resolves in 1 - 2 days.

Reporting of suspected adverse reactions
Reporting suspected adverse reactions after authorisation of the medicinal product is important. It allows continued monitoring of the benefit/risk balance of the medicinal product. Healthcare professionals are asked to report any suspected adverse reactions via the national reporting system listed.
You can also report side effects to the manufacturer (See section 7).

4.9 Overdose

An overdose of the single-dose vaccine is unlikely to occur.
It is recommended to carefully review the medical history of the patient with respect to previous immunizations in order to avoid the administration in recently immunized or re-immunized individuals containing tetanus and diphtheria toxoid.

5. PHARMACOLOGICAL PROPERTIES

5.1 Pharmacodynamic properties

Pharmacotherapeutic group: Antiinfectives for systemic use, vaccines, bacterial vaccines, tetanus vaccines, tetanus toxoid, combination with diphtheria toxoid.
ATC code: J07AM51

Mechanism of action
TETADIF is a suspension of purified and adsorbed tetanus and diphtheria toxoid onto aluminum adsorbent, prepared from toxins of *Corynebacterium diphtheriae* and *Clostridium tetani* by detoxification followed by purification. The vaccine contains a reduced quantity of the diphtheria antigen. After vaccination, antibodies are produced against the two antigens of the vaccine, providing protection against diphtheria and tetanus. The immunity is strengthened after re-immunization and is considered to last from 5 to 10 years.

Efficiency against diphtheria
The diphtheria toxoid provides protection against diphtheria.
Immunoprophylaxis is the only way to prevent diphtheria. It is performed with diphtheria toxoid, most often combined with tetanus toxoid or killed pertussis bacteria. Children aged between three months to 8 years are subject to vaccination although in recent years, people up to the age of 35 have also been immunized. The mass application of the vaccine in our country has led to the elimination of any incidence of diphtheria in Bulgaria. The data indicated in Table 1 about the immunological status of the population demonstrated that children and persons under 15 years of age show the highest level of protection against diphtheria. The increasing of age leads to decrease in the percentage of protected persons, especially in the adults over 45. However, the percentage of people protected from diphtheria between the ages of 16 and 65 is higher than the rates from research in some European countries and the United States. For example, in Sweden about 56.9% of the population lacks protection against diphtheria, in Germany, this percentage is about 52.2%, and in Denmark about 36%.

Table 1: Evaluation of the immune status of the population in Bulgaria against diphtheria in different age groups.

Age group	% serums with full protection (titer > 0.1 IU)	% serums with basic immunity (titer 0.01 to 0.09 IU)	% serums with no protection (titer < 0.009 IU)
**Children aged 0 to 7 years**	94.45	-	5.55
**Persons aged 8 to 15 years**	100	-	-
**Persons aged 16 to 25 years**	81.39	2.9	15.71
**Persons aged 26 to 35 years**	78.69	3.6	17.71
**Persons aged 36 to 45 years**	77.0	1.0	22.0
**Persons aged 46 to 55 years**	61.92	1.58	36.5
**Persons between 56 to 65 years**	61.64	7.6	30.76
**Adults aged over 65 years**	11.77	-	88.23

**The results were obtained by ELISA: Enzyme-linked immunoassay;*

Efficiency against tetanus
Tetanus toxoid provides protection against tetanus.

One of the means of treatment tetanus is a specific immune prophylaxis with tetanus toxoid. In human organism it causes formation of specific antibodies, which play role in protection against tetanus. The immunity in tetanus is conditioned and depends on the quantity and possibility of the specific antibodies in short time to neutralize the tetanus toxin.
Tetanus still represents a health problem for many countries around world mainly due to its high mortality.
In Bulgaria the morbidity and mortality from the tetanus were high. Since 1959, obligatory specific immune prophylaxis against tetanus was introduced in Bulgaria, gradually covering the entire population. This led to a significant decrease in disease incidence (from 2.7 %000 in 1959 to 0.01 %000 in 1998). This proves the good protective effect of tetanus toxoid and the optimal scheme of administration, regardless of the age. In comprehensive clinical studies in persons with chronic diseases like allergies, diabetes, rheumatism, uro-genital diseases and persons over 70 years of age, showed good tolerance for these risk group of patients, witch allows their inclusion in the obligatory immunization schedule.

Seroepidemiological studies conducted in recent years showed the highest antitoxic titer among children and adolescent because they are included in the obligatory immunization schedule. Good immunity is established in the other age groups with slight decrease of protective titer, in relation to an insufficient population density. Single cases of disease in these age groups are observed. The observations on tetanus toxoid administration in over 2 000 persons in the last ten years, reveals that it is not reactogenic.

5.3 Preclinical safety data

Non-clinical data reveal no special hazard for humans.

6. PHARMACEUTICAL PARTICULARS

6.1 List of excipients

in single dose ampoule:
Aluminium hydroxide
Sodium chloride
Water for injection

in multi-dose vials:
Aluminium hydroxide
Thiomersal
Sodium chloride
Water for injection

6.2 Incompatibilities

In the absence of compatibility studies, this medicinal product must not be mixed with other medicinal products.

In concomitant administration with other injectable medicinal products, each individual product needs to be drawn up and administered using a separate syringe and needle, and at a site different than the vaccine injection site.

6.3 Shelf life

3 years

6.4 Special precautions for storage

Store and transport refrigerated (2°C – 8°C).
Store in the original carton package.
Do not freeze!
A FROZEN VACCINE IS UNFIT FOR USE!
Keep this medicine out of the sight and reach of children.
Use the vaccine in the multi-dose vial immediately after withdrawing the first dose!
Any unused vaccine should be discarded!

6.5 Nature and contents of container

Clear, colourless, flip-off glass ampoule (type I).
The ampoules contain a vaccine in a volume of 0.5 ml – 1 dose.
Pack size: carton box of 1, 10 or 50 ampoules.

Clear, colourless, glass vial (type I).
The vial contains vaccine in volume of 5.0 ml - 10 doses.
The vial contains vaccine in volume of 10.0 ml - 20 doses.
Pack size: carton boxes of 10 vials.

Not all pack sizes may be marketed.

6.6 Special precautions for disposal and other handling

The precipitated vaccine is a clear, colourless supernatant fluid with whitish precipitate.

FROZEN VACCINE IS UNFIT FOR USE!
Do not administer intravenously.
Do not use expired vaccine.
Do not use in case the integrity of the ampoule (vial) is compromised or the labelling is unclear/ deleted.

Any unused medicinal product or waste material should be disposed of in accordance with local requirements.

7. MARKETING AUTHORISATION HOLDER

BB - NCIPD Ltd.
26, Yanko Sakazov Blvd.
1504 Sofia
Bulgaria
Tel. +359 2 944 61 91
Fax +359 2 943 34 55
E-mail: bulbio@bulbio.com

8. MARKETING AUTHORISATION NUMBER(S)

II-15236/ 11 October 2011

Registration No 20011159

9. DATE OF FIRST AUTHORISATION/RENEWAL OF THE AUTHORISATION

Date of first authorisation: 28 November 2001
Date of latest renewal: 11 October 2011

10. DATE OF REVISION OF THE TEXT

April 2022

TECHNICAL DATA SHEET

1. NAME OF THE MEDICINAL PRODUCT

diTeBooster, injectable suspension in single-dose vials or prefilled single-dose syringe.

Diphtheria and tetanus vaccine (absorbed, reduced antigen(s) content).

2. QUALITATIVE AND QUANTITATIVE COMPOSITION

One dose (0.5 ml) contains:

Purified diphtheria toxoid[1]	6.25 Lf / ≥ 2 IU
Purified tetanus toxoid[1]	6.25 Lf / ≥ 20 IU

[1] Absorbed in hydrated aluminium hydroxide (AL(OH)$_3$), corresponding to 0.5 mg of aluminium (Al^{3+}).

The diphtheria and tetanus toxins, obtained from cultures of *Corynebacterium diphtheriae* and *Clostridium tetani*, are purified and detoxified.

No substance of human origin is used during the manufacture of the vaccine.

The vaccine may contain traces of formaldehyde that is used during the manufacturing process (see section 4.4).

For the full list of excipients, see section 6.1.

3. PHARMACEUTICAL FORM

Suspension for injection in single-dose vials or pre-filled syringe (injection). Colourless

or light yellow suspension of white/grey particles.

4. CLINICAL DATA

4.1. Therapeutic indications

Active immunization against tetanus and diphtheria in persons from 5 years of age.

Revaccination against diphtheria and tetanus, and primary immunization of persons with missing, incomplete or unknown primary immunization.

Tetanus prophylaxis in individuals 5 years of age and older with tetanus-prone lesions and concurrent diphtheria immunization.

Use of diTeBooster should be in accordance with official national recommendations.

4.2. Dosage and form of administration

MARÍA CASANI FERNÁNDEZ DE NAVARRETE
Traductor/a Intérprete Jurado/a de INGLÉS
Nº 3692

<u>Dosage</u>

diTeBooster should be administered intramuscularly as a single 0.5 ml dose for all ages.

Revaccination
diTeBoost can be used for revaccination of persons who have been previously vaccinated against diphtheria and tetanus according to national recommendations.

An enhanced response can only be expected in persons with primary vaccination.

Repeated vaccination against diphtheria and tetanus should be carried out at intervals according to official recommendations (usually 10 years).

Primary immunisation
Persons with missing, incomplete, or unknown primary immunization may be vaccinated with diTeBooster. More than one vaccine may be needed to achieve protective immunity against diphtheria and tetanus. National recommendations should be followed.

Tetanus-prone lesions
In people with tetanus-prone lesions, diTeBooster may be given when diphtheria vaccination is also relevant. Tetanus immunoglobulin can be administered simultaneously according to national recommendations.

Pediatric population
Safety and efficacy of diTeBooster in children under the age of 5 years has not been established. No data available.

Method of administration

diTeBooster should be administered intramuscularly (IM), preferably in the deltoid region.

Do not inject intravascularly.

Shake before use.

In certain indications (e.g. hemorrhagic diathesis) diTeBooster can be administered deep subcutaneously.

4.3. Contraindications

Serious adverse reactions after previous vaccination with the vaccine or hypersensitivity to the active ingredient(s), or to any of the excipients included in section 6.1.

4.4. Special warnings and precautions for use

- As with all injectable vaccines, adequate treatment and medical supervision should always be available in the event of a rare anaphylactic episode following vaccine administration.
- Vaccination should be postponed in case of acute illness with fever.
- Under no circumstances should diTeBooster be administered intravascularly.
- As with any injectable vaccine, diTeBooster should be administered with caution to persons with uncontrolled coagulopathy, as bleeding may occur following intramuscular administrations.
- In individuals with compromised immune response, the serological response may be altered. Vaccination may be carried out in persons receiving immunosuppressive therapy, although a decreased immune response may occur.
- The final product may contain traces of formaldehyde that is used during the process of

MARÍA CASANI FERNÁNDEZ DE NAVARRETE
Traductora-Intérprete Jurado/a de INGLÉS
Nº 3692

MINISTERIO DE SANIDAD POLÍTICA SOCIAL E IGUALDAD
Agencia española de medicamentos y

manufacturing. Caution should be exercised in subjects with known hypersensitivity to formaldehyde.

- diTeBooster contains less than 1 mmol sodium (23 mg) per dose, i.e. essentially 'sodium-free'.
- Too frequent booster vaccination will increase the risk of adverse reactions.

Traceability
In order to improve the traceability of biological drugs, the name and batch number of the drug administered should be clearly recorded.

4.5. Interaction with other drugs and other forms of interaction

Do not mix with other vaccines in the same syringe or vial.

The concomitant use of diTeBooster with other inactivated vaccines has not been studied. Co-administration is unlikely to result in interference with immune responses. When deemed necessary, diTeBooster can be administered simultaneously with other vaccines, at a different injection site.

If immediate protection is needed, diTeBooster can be given at the same time as tetanus immunoglobulin. Injections of diTeBooster and tetanus immunoglobulin should be done in separate limbs.

4.6. Fertility, pregnancy and breastfeeding

Pregnancy

No animal data are available. In humans, data are inadequate to assess the risk of teratogenicity or phototoxicity during pregnancy.
During pregnancy, the potential risk of clinical infection after exposure should be assessed against the theoretical risks of the vaccine.

Breastfeeding

There is no indication that vaccination of nursing mothers with diTeBooster is harmful to the child. Fertility

The effect on reproductive organs has not been studied in developmental toxicology studies. However, there is no indication that vaccination has an effect on male and female fertility.

4.7. Effects on the ability to drive and use machines

The influence of diTeBooster on the ability to drive and use machines is null or negligible.

4.8. Adverse reactions

In relation to the administration of diTeBooster, the most common adverse reactions are: Redness and swelling at the injection site and fever. Reactions usually start within 48 hours of vaccination.

List in table form of adverse reactions.
The adverse reactions listed below are based on data from clinical trials in children, adolescents, and adults, and are classified according to MedDRA system organ class terminology.
The safety evaluation of diTeBooster also includes adverse reactions from clinical trials and spontaneous reports with vaccines containing the same or higher content of diphtheria and tetanus antigens than diTeBooster in combination with aluminum hydroxide and other vaccine antigens.

MARÍA CASANI FERNÁNDEZ DE NAVARRETE
Traductor/a Intérprete Jurado/a de INGLÉS
Nº 3692

Classification by organs, systems and frequency	Adverse reactions
**Disorders of the immunological system**<br>Rare ≥ 1/10 000 and < 1/1 000	Hypersensitivity, including anaphylactic reactions.
**Nervous system disorders**<br>Very frequent (≥1/10)<br>Common (≥ 1/100 and <1/10)<br>Very rare < 1/10 000	Headache.<br>Dizziness<br>Vasovagal syncope
**Gastrointestinal disorders**<br>Common (≥ 1/100 and <1/10)	Nausea, vomiting and diarrhoea
**Skin and subcutaneous tissue disorders**<br>Uncommon (> 1/ 1 000 and < 1/100)<br>Rare ≥ 1/10 000 and < 1/1 000	Eczema and dermatitis<br>Urticarial reactions
**Musculoskeletal and connective tissue disorders Vascular**<br>Common (≥ 1/100 and <1/10)	Myalgia
**General disorders and alterations at the site of administration**<br>Very frequent (≥1/10)<br><br><br><br>Common (≥ 1/100 and <1/10)<br><br><br><br>Rare ≥ 1/10 000 and < 1/1 000	Redness / Swelling at the injection site*<br>Pain at the site of injection<br>Injection site itching<br>Fatigue<br>Malaise<br>Fever ≥ 38°C<br>Redness/ Swelling ≥ 5 cm at the site of the injection<br>High fever > 40 °C<br>Sterile granuloma or abscess at the injection site.

*Less frequent redness/swelling at the injection site has been observed in adults.

Pediatric population
The safety evaluation of diTeBooster studied in clinical trials includes children aged 4 years and older and adolescents

Reporting of suspected adverse reactions:

Reporting suspected adverse reactions after authorisation of the drug is important. It allows continued monitoring of the benefit/risk balance of the drug. Healthcare professionals are invited to report suspected adverse reactions through the Spanish Pharmacovigilance System for Medicinal Products for Human Use: www.notificaRAM.es

4.9. Overdose

No cases of overdose have been reported.

MARÍA CASANI FERNÁNDEZ DE NAVARRETE
Traductor/a Intérprete Jurado/a de INGLÉS
Nº 3692

5. PHARMACOLOGICAL PROPERTIES

5.1. Pharmacodynamic properties

Pharmacotherapeutic group: Tetanus, toxoid, combinations with diphtheria toxoid.
ATC code: J07AM51.

Action mechanism

Antibodies to vaccine antigens are produced shortly after vaccination. Protection against diphtheria and tetanus is expected to last for at least 10 years

Clinical efficacy and safety

Clinical trials have been conducted with diTeBooster in children, adolescents and adults. The immunogenicity assessment of diTeBooster also includes data from clinical trials with vaccines containing the same content of diphtheria and tetanus antigens as diTeBooster in combination with aluminum hydroxide and other vaccine antigens, e.g., acellular pertussis. The results are summarised in the following table.

Study population	Age	Children 5-6 years	Children 10 years	Teenagers from 14-15 years old	Adults 18-55 years old
	Vaccination history.	3 x DTaP First year of life	3 x DT First year of life	3 x DTaP First year of life 1 x TdaP 4-6 years old.	3-4 x D and T First year of life
Antigen	Immune system				
Diphtheria	≥ 0.1 IU/mL	98.6 -100%	-	100%	98.5-100%
	≥ 0.01 IU/ml	100%	100%	-	98.8-100%
Tetanus	≥ 0.1 IU/mL	99.3-100%	100%	100%	99.4- 100 %
	≥ 0.01 IU/ml	99.3-100%	100%	-	99.4- 100 %

Antibodies to diphtheria and tetanus were measured one month after vaccination.

Diphtheria and tetanus antibody levels of ≥ 0.01 IU/ml are considered the minimum level of antibody required to confer some degree of protection, while levels of at least 0.1 IU/ml are considered protective.

5.2. Pharmacokinetic properties

Evaluation of the pharmacokinetic properties for vaccines is not necessary.

5.3. Preclinical safety data

Subacute and acute toxicity of vaccine components have been studied in animals. No clinical symptoms or systemic toxicity have been recorded.

6 . PHARMACEUTICAL DATA

6.1. List of excipients

Per dose = 0.5 ml:
Sodium hydroxide until pH = 7
Sodium chloride

MARÍA CASANI FERNÁNDEZ DE NAVARRETE
Traductor/a Intérprete Jurado/a de INGLÉS
Nº 3692

Water for injectable preparations.

The pH of the vaccine is about 7.

For absorbents, see section 2.

6.2. Incompatibilities

In the absence of compatibility studies, this vaccine must not be mixed with other vaccines or drugs.

6.3. Validity period

3 years.

6.4. Special storage precautions

Store in a refrigerator (2°C to 8°C).
Do not freeze.
Discard if vaccine has been frozen.

6.5. Nature and content of the packaging

Single-dose vials (type I glass) closed with a stopper (chlorobutyl rubber) containing 0.5 ml (1 dose).
Package size: 1 x 0.5 ml, 5 x 0.5 ml and 10 x 0.5 ml.
The vial caps do not contain latex.

Single-dose pre-filled syringe (type I glass) with 0.5 ml (1 dose).
Package size: 1 x 0.5 ml, 5 x 0.5 ml, 10 x 0.5 ml and 20 x 0.5 ml.
Only some package sizes may be commercially available.

6.6. Special precautions for disposal and further handling

Shake before use.

After thorough resuspension, the vaccine should show up as a colorless or light yellow suspension of white and gray particles.

Examine the vaccine for foreign particles and/or discoloration before use. If these conditions are met, the product should not be administered

Disposal of the unused drug and all materials that have been in contact with it should be carried out in accordance with local regulations.

7. MARKETING AUTHORIZATION HOLDER

AJ vaccines A/S
Artillerivej 5
DK-2300 Copenhagen S
Denmark

MARÍA CASANI FERNÁNDEZ DE NAVARRETE
Traductor/a Intérprete Jurado/a de INGLÉS
Nº 3692

8. MARKETING AUTHORISATION NUMBER(S)

Pre-filled syringe: 68579

MINISTERIO DE SANIDAD, POLÍTICA SOCIAL E IGUALDAD
Agencia española de medicamentos y

9. DATE OF THE FIRST AUTHORIZATION/ RENEWAL OF THE AUTHORIZATION

Date of first authorisation: Pre-filled syringe: 28/March/2007
Date of last renewal: Pre-filled syringe: 25/May/2010

10. DATE OF REVISION OF THE TEXT

01/2024

Don/Doña MARÍA CASANI FERNÁNDEZ DE NAVARRETE, Traductor/a-Intérprete Jurado/a de nombrado/a por el Ministerio de Asuntos Exteriores y de Cooperación, CERTIFICA que lo que ante traducción fiel y completa al inglés de un documento redactado en español.

Mr/Ms. MARÍA CASANI FERNÁNDEZ DE NAVARRETE, Official English Translator/Interpreter of appointed by the Spanish Ministry of Foreign Affairs and Cooperation, HEREBY CERTIFIES that the fore an accurate and complete translation to English of the original document, written in Spanish.

En Madrid a dieciséis de diciembre de dos mil veinticuatro.

MARÍA CASANI FERNÁNDEZ DE NAVARRETE
Traductor/a Intérprete Jurado/a de INGLÉS
Nº 3692

FICHA TÉCNICA

1. NOMBRE DEL MEDICAMENTO

diTeBooster, suspensión inyectable en viales monodosis o jeringa monodosis precargada.

Vacuna contra la difteria y el tétanos (adsorbida, contenido en antígeno(s) reducido).

2. COMPOSICIÓN CUALITATIVA Y CUANTITATIVA

Una dosis (0,5 ml) contiene:

Toxoide diftérico purificado[1]	6,25 Lf / $\geq$ 2 UI
Toxoide tetánico purificado[1]	6,25 Lf / $\geq$ 20 UI

[1] Absorbido en hidróxido de aluminio hidratado ($AL(OH)_3$), correspondiente a 0,5 mg de aluminio (Al^{3+}).

Las toxinas difetérica y tetánica, obtenidas a partir de cultivos de *Corynebacterium diphtheriae* y *Clostridium tetani*, se purifican y detoxifican.

Durante la fabricación de la vacuna no se utiliza ninguna sustancia de origen humano.

La vacuna puede contener trazas de formaldehido que se utiliza durante el proceso de fabricación (ver sección 4.4).

Para consultar la lista completa de excipientes ver sección 6.1.

3. FORMA FARMACÉUTICA

Suspensión inyectable en viales monodosis o jeringa precargada (inyectable).

Suspensión incolora o amarilla clara de partículas blancas/grises.

4. DATOS CLÍNICOS

4.1. Indicaciones terapéuticas

Inmunización activa contra el tétanos y la difteria en individuos a partir de 5 años de edad.

Revacunación contra la difteria y el tétanos, e inmunización primaria de individuos con inmunización primaria faltante, incompleta o desconocida.

Profilaxis del tétano en individuos a partir de 5 años de edad con lesiones propensas al tetanos y con inmunización simultaneo contra la difteria.

El uso de diTeBooster debe realizarse siguiendo las recomendaciones oficiales nacionales.

4.2. Posología y forma de administración

Posología

diTeBooster debe administrase por vía intramuscular en forma de una dosis única de 0,5 ml para todas las edades.

MARÍA CASAN FERNÁNDEZ DE NAVARRETE
Traductor/a Intérprete Jurado/a de INGLÉS
Nº 3692

Revacunación
diTeBoost puede ser utilizado para la revacunación de individuos que han sido previamente vacunados contra la difteria y el tétanos según las recomendaciones nacionales.

Solo se puede esperar una respuesta potenciada en los individuos con vacunación primaria.

La vacunación repetida frente a la difteria y el tétanos debe realizarse a intervalos según las recomendaciones oficiales (generalmente 10 años).

Inmunización primaria
Los individuos con inmunización primaria faltante, incompleta o desconocida pueden ser vacunados con diTeBooster. Es posible que se necesite más de una vacuna para conseguir una inmunidad protectora contra la difteria y el tétanos. Deben seguirse las recomendaciones nacionales.

Lesiones propensas al tétanos
En las personas con lesiones propensas al tétanos, se puede administrar diTeBooster cuando la vacunación contra la difteria también es pertinente. La inmunoglobulina del tétanos puede administrarse simultáneamente de acuerdo a las recomendaciones nacionales.

Población pediátrica
No se ha establecido la seguridad y eficacia de diTeBooster en niños menores de 5 años. No se dispone de datos.

Forma de administración

diTeBooster debe administrarse por vía intramuscular (IM), preferiblemente en la región del deltoides.

No se debe inyectar por vía intravascular.

Agitar antes de usar.

En ciertas indicaciones (por ejemplo, diátesis hemorrágica) se puede administrar diTeBooster por vía subcutánea profunda.

4.3. Contraindicaciones

Reacciones adversas graves tras vacunación anterior con la vacuna o hipersensibilidad al (a los) principio(s) activo(s), o a alguno de los excipientes incluidos en la sección 6.1.

4.4. Advertencias y precauciones especiales de empleo

- Al igual que todas las vacunas inyectables siempre debe disponerse de un tratamiento y una supervisión médica adecuados en caso de que se produzca un episodio anafiláctico poco frecuente después de la administración de la vacuna.
- La vacunación debe posponerse en caso de enfermedad aguda con fiebre.
- Bajo ninguna circunstancia debe administrarse diTeBooster por via intravascular.
- Como con cualquier vacuna inyectable, diTeBooster debe administrarse con precaución a los individuos con coagulopatía no controlada, ya que puede producirse una hemorragia tras las administraciones intramusculares.
- En individuos con la respuesta inmunitaria comprometida, puede alterarse la respuesta serológica. Puede llevarse a cabo la vacunación en individuos que reciban tratamiento inmunosupresor, aunque puede producirse una menor respuesta inmunológica.
- El producto final puede contener trazas de formaldehído que se utiliza durante el proceso de

Traductor/a Intérprete Jurado/a de INGLES Nº 3692

fabricación. Debe tenerse precaución en sujetos con hipersensibilidad conocida al formaldehído.

- diTeBooster contiene menos de 1 mmol de sodio (23 mg) por dosis; esto es, esencialmente "exento de sodio".
- Una vacunación de refuerzo demasiado frecuente aumentará el riesgo de reacciones adversas.

Trazabilidad

Con objeto de mejorar la trazabilidad de los medicamentos biológicos, el nombre y el número de lote del medicamento administrado deben estar claramente registrados.

4.5. Interacción con otros medicamentos y otras formas de interacción

No mezclar con otras vacunas en la misma jeringa o vial.

No se ha estudiado el uso concomitante de diTeBooster con otras vacunas inactivadas. Es improbable que la coadministración pueda originar una interferencia con las respuestas inmunitarias. Cuando se considere necesario, diTeBooster puede administrarse simultáneamente con otras vacunas, en un lugar de inyección distinto.

Si es necesario proporcionar una protección inmediata, se puede administrar diTeBooster al mismo tiempo que la inmunoglobulina del tétanos. Las inyecciones de diTeBooster y la inmunoglobulina del tétanos deben hacerse en miembros separados.

4.6. Fertilidad, embarazo y lactancia

Embarazo

No se dispone de datos relevantes en animales. En humanos, los datos son inadecuados para evaluar el riego de teratogenicidad o de fototoxicidad durante el embarazo.
Durante el embarazo debe evaluarse el posible riesgo de infección clínica después de la exposición frente a los riesgos teóricos de la vacuna.

Lactancia

No hay indicios de que la vacunación de madres lactantes con diTeBooster sea perjudicial para el niño.

Fertilidad

El efecto sobre los órganos reproductores no se ha estudiado en estudios toxicológicos del desarrollo. Sin embargo, no hay indicios de que la vacunación tenga un efecto sobre la fertilidad masculina y femenina.

4.7. Efectos sobre la capacidad para conducir y utilizar máquinas

La influencia de diTeBooster sobre la capacidad para conducir y utilizar máquinas es nula o insignificante.

4.8. Reacciones adversas

En relación con la administración de diTeBooster, las reacciones adversas más frecuentes son:
Enrojecimiento e hinchazón en el lugar de la inyección y fiebre. Las reacciones se inician habitualmente durante las 48 horas posteriores a la vacunación.

Lista tabulada de reacciones adversas.

Las reacciones adversas que se enumeran a continuación se basan en datos de ensayos clínicos en niños, adolescentes y adultos, y se clasifican según la terminología MedDRA de clasificación por órganos y sistemas.
La evaluación de seguridad de diTeBooster también incluye reacciones adversas de los ensayos clínicos e informes espontáneos con vacunas que contienen el mismo o mayor contenido de antígenos de la difteria y el tétanos que diTeBooster en combinación con hidróxido de aluminio y otros antígenos de la vacuna.

MARÍA CASANI FERNÁNDEZ DE NAVARRETE
Traductor/a Intérprete Jurado/a de INGLÉS
Nº 3692

Maria Casa

MINISTERIO DE SANIDAD, POLÍTICA SOCIAL E IGUALDAD
Agencia española de medicamentos y productos sanitarios

Clasificación por órganos, sistemas y frecuencia	Reacciones adversas
**Trastornos del sistema inmunológico**	
Raras (≥1/10 000 y <1/1 000)	Hipersensibilidad, incluyendo reacciones anafilacticas.
**Trastornos del sistema nervioso**	
Muy frecuentes (≥1/10)<br>Frecuentes (≥1/100 y <1/10)<br>Muy raras (<1/10 000)	Dolor de cabeza.<br>Mareos<br>Síncope vasovagal
**Trastornos gastrointestinales**	
Frecuentes (≥1/100 y <1/10)	Nausea, vomito y diarrea
**Trastornos de la piel y del tejido subcutáneo**	
Poco frecuentes (≥1/1 000 y <1/100)<br>Raras (≥1/10 000 y <1/1 000)	Eccema y dermatitis<br>Reacciones de urticaria
**Trastornos musculoesqueléticos y del tejido conjuntivo**	
Frecuentes (≥1/100 y <1/10)	Mialgia
**Trastornos generales y alteraciones en el lugar de administración.**	
Muy frecuentes (≥1/10)	Enrojecimiento / Hinchazón en el lugar de la inyección*<br>Dolor en el lugar de la inyección<br>Picazón en el lugar de la inyección<br>Fatiga
Frecuentes (≥1/100 y <1/10)	Malestar<br>Fiebre ≥ 38 °C<br>Enrojecimiento/ Hinchazón ≥ 5 cm en el lugar de la inyección
Raras (≥1/10 000 y <1/1 000)	Fiebre alta > 40 °C<br>Granuloma o abceso esteril en el lugar de la inyección.

*En los adultos se ha observado un enrojecimiento/hinchazón menos frecuente en el lugar de la inyección.

Población pediátrica
La evaluación de seguridad de diTeBooster estudiado en los ensayos clínicos incluye a niños a partir de 4 años y adolescentes

Notificación de sospechas de reacciones adversas:

Es importante notificar sospechas de reacciones adversas al medicamento tras su autorización. Ello permite una supervisión continuada de la relación beneficio/riesgo del medicamento. Se invita a los profesionales sanitarios a notificar las sospechas de reacciones adversas a través del Sistema Español de Farmacovigilancia de medicamentos de Uso Humano: www.notificaRAM.es.

4.9. Sobredosis

No se han registrado casos de sobredosis.

MARÍA CASANI FERNÁNDEZ DE NAVARRETE
Traductor/a Intérprete Jurado/a de INGLÉS
Nº 3692

5. PROPIEDADES FARMACOLÓGICAS

5.1. Propiedades farmacodinámicas

Grupo farmacoterapéutico: Tetánico, toxoide, combinaciones con toxoide diftérico.
Código ATC: J07AM51.

Mecanismo de acción

Poco después de la vacunación se producen anticuerpos contra los antígenos de la vacuna. Se espera una protección contra la difteria y el tétanos con una duración de al menos 10 años

Eficacia clínica y seguridad

Se han realizado ensayos clínicos con diTeBooster en niños, adolescentes y adultos. La evaluación de la inmunogenicidad de diTeBooster también incluye datos de ensayos clínicos con vacunas que contienen el mismo contenido de antígenos de la difteria y el tétanos que diTeBooster en combinación con hidróxido de aluminio y otros antígenos de la vacuna, por ejemplo, la tos ferina acelular. Los resultados se resumen en el cuadro que figura a continuación.

Población dc estudio	Edad	Niños de 5-6 años	Niños de 10 años	Adolescentes de 14-15 años	Adultos de 18-55 años
	Historial de vacunación.	3 x DTaP Primer año de vida	3 x DT Primer año de vida,	3 x DTaP Primer año de vida<br>1 x TdaP 4-6 años de edad	3-4 x D and T Primer año de vida
Antígeno	Respuesta inmune				
Difteria	≥ 0,1 UI/ml	98,6-100 %	-	100 %	98,5-100 %
	≥ 0,01 UI/ml	100 %	100 %	-	98,8-100 %
Tétanos	≥ 0,1 UI/ml	99,3-100 %	100 %	100 %	99,4-100 %
	≥ 0,01 UI/ml	99,3-100 %	100 %	-	99,4-100 %

Los anticuerpos contra la difteria y el tétanos se midieron un mes después de la vacunación.

Los niveles de anticuerpos contra la difteria y el tétanos de ≥ 0,01 UI/ml se consideran el nivel mínimo de anticuerpos requerido para conferir cierto grado de protección, mientras que los niveles de al menos 0,1 UI/ml se consideran protectores.

5.2. Propiedades farmacocinéticas

La evaluación de las propiedades farmacocinéticas para las vacunas no es necesaria.

5.3. Datos preclínicos sobre seguridad

Se han estudiado en animales la toxicidad subaguda y aguda de los componentes de la vacuna. No se han registrado síntomas clínicos ni toxicidad sistémica.

6 . DATOS FARMACÉUTICOS

6.1. Lista de excipientes

Por dosis = 0,5 ml:
Hidróxido de sodio hasta pH = 7
Cloruro de sodio

MARÍA CASANI FERNÁNDEZ DE NAVARRETE
Traductor/a Intérprete Jurado/a de INGLÉS
Nº 3692

Agua para preparaciones inyectables.

El pH de la vacuna es aproximadamente 7.

Para los adsorbentes, ver la sección 2.

6.2. Incompatibilidades

En ausencia de estudios de compatibilidad, esta vacuna no debe mezclarse con otras vacunas ni medicamentos.

6.3. Periodo de validez

3 años.

6.4. Precauciones especiales de conservación

Conservar en nevera (entre 2 °C y 8 °C).
No congelar.
Desechar si la vacuna ha sido congelada.

6.5. Naturaleza y contenido del envase

Viales monodosis (vidrio tipo I) cerrados con un tapón (goma de clorobutilo) que contiene 0,5 ml (1 dosis).
Tamaño del envase: 1 x 0,5 ml, 5 x 0,5 ml y 10 x 0,5 ml.
Los tapones de los viales no contienen látex.

Jeringa monodosis precargada (vidrio tipo I) con 0,5 ml (1 dosis).
Tamaño del envase: 1 x 0,5 ml, 5 x 0,5 ml, 10 x 0,5 ml y 20 x 0,5 ml.
Puede que solamente estén comercializados algunos tamaños de envases.

6.6. Precauciones especiales de eliminación y otras manipulaciones

Agitar antes de usar.

Tras la resuspensión a fondo, la vacuna debe mostrarse como una suspensión incolora o amarilla clara de partículas blancas y grises.

Examine la vacuna para ver si hay partículas extrañas y/o decoloración antes de usarla. Si se dan estas condiciones, el producto no debe ser administrado

La eliminación del medicamento no utilizado y de todos los materiales que hayan estado en contacto con él, se realizará de acuerdo con la normativa local.

7. TITULAR DE LA AUTORIZACIÓN DE COMERCIALIZACIÓN

AJ Vaccines A/S
Artillerivej 5
DK-2300 Copenhague S
Dinamarca

8. NÚMERO(S) DE AUTORIZACIÓN DE COMERCIALIZACIÓN

MARÍA CASANI FERNÁNDEZ DE NAVARRETE
Traductor/a Intérprete Jurado/a de INGLÉS
Nº 3692

Jeringa precargada: 68579

9. FECHA DE LA PRIMERA AUTORIZACIÓN/ RENOVACIÓN DE LA AUTORIZACIÓN

Fecha de la primera autorización: Jeringa precargada: 28/marzo/2007
Fecha de la última renovación: Jeringa precargada: 25/mayo/2010

10. FECHA DE LA REVISIÓN DEL TEXTO

01/2024

MARÍA CASANI FERNÁNDEZ DE NAVARRETE
Traductor/a Intérprete Jurado/a de INGLÉS
Nº 3692

9. ELISA Method: Anti-Diphtheria Toxoid and Anti-Tetanus Toxoid

Anti-Diphtheria toxoid and Tetanus toxoid IgG concentrations in serum were measured using validated enzyme linked immunosorbent assays (ELISAs). Antibody concentration were measured relative to the International Standard for Tetanus Immunoglobulin human (NIBSC product number 26/488) or the International Standard for Diphtheria Antitoxin Human (NIBSC product number 10/262). Nunc® MaxiSorp™ plates were coated with either Diphtheria toxoid or Tetanus toxoid antigen by overnight incubation at 2-8°C, with antigen diluted in 15 mM sodium carbonate, 35 mM sodium bicarbonate buffer, pH9.6. The next day the coated plates were washed five times with 300µl of phosphate buffered saline containing 0.1%(v/v) Tween 20 (PBST) and blocked with PBST containing 5% (v/v) foetal bovine serum (FBS, Sigma Aldrich) for 60 min at 20 °C ±2 °C. Test samples, standard serum and quality control (QC) serum dilutions were prepared in PBST, 5%(v/v) FBS in a separate dilution plate (Sterilin™) using a starting dilution of 1/125 and seven further three-fold serial dilutions down the plate. The blocking buffer from the coated plates was removed and 100µl/well of each serial dilution from the dilution plates was transferred to the coated plate. Plates were incubated with sera for 60 min±10min at 20 °C±2 °C to facilitate antibody binding and then washed five times with 300µl of PBST to wash away unbound antibodies. Bound antibody was detected using a goat anti-human IgG Fcγ antibody conjugated to alkaline phosphatase (Jackson ImmunoResearch), diluted in PBST to 1/5000, and incubated with 100µl/well for 60 min+10min at 20 °C±2 °C. After washing five times with PBST, 100µl/well of Bio FX™ AP yellow substrate (p-nitrophenyl phosphate, Surmodics Inc.) was added and plates were incubated for 60 min±10min at 20 °C ±2 °C after which time the reaction was stopped by the addition of 50µl/well of 3M sodium hydroxide. Five minutes after stopping the reaction, the optical density (OD, A405nm-A690nm) of each well was measured on a VersaMax™ plate reader using Softmax® Pro GxP software customised protocols. The protocols were validated are designed to process the raw data by fitting an unweighted four parameter logistic curve to the standard serum dose response data on each plate. OD values from each serial dilution for each test sample and QC serum were used to interpolate concentrations from the standard dose response curve run on the same plate and a mean of acceptable values for each sample was calculated. System suitability criteria (for the assay plate and test sample) were applied and reportable values were assigned following defined rules to meet data integrity criteria.

Annex 12

Diphtheria antitoxin assay in Vero cell (in vitro toxin neutralization)

The method described here involves staining for cell viability with the yellow tetrazolium salt (MTT), adapted from NIBSC method (see below link).

A12.1 MATERIALS

12.1.1 CRITICAL REAGENTS AND STANDARDS

- Diphtheria antitoxin: 1st WHO International Standard for diphtheria antitoxin, equine (DI). or the 1st WHO International Standard for Diphtheria Antitoxin Human (10/262) are available from NIBSC (https://www.nibsc.org/science_and_research/bacteriology/diphtheria.aspx)
- In-house standards or a panel of positive control human serum samples of different level of defined activities in IU (e.g. 0.1, 0.01, 0.001 IU/ml) may be included to monitor assay performance;
- Diphtheria toxin: A purified preparation of diphtheria toxin of defined activity (minimum cytopathic dose) and stability should be used;
- Vero cells: Vero cells are available on request from Chief, Biologicals, WHO, Geneva, Switzerland. Cells may also be obtained from culture collections (ATCC, EDQM) or other sources provided that their robustness and sensitivity to diphtheria toxin is known.

12.1.2 EQUIPMENT

- Laminar airflow cabinet
- Incubator (+36 to +38oC)
- Flat bottomed sterile 96 well tissue culture plates
- Multichannel manual or electronic pipettes 50-200 µl
- Sterile tips for pipettes
- Sterile serological pipettes (5-25 ml) and electronic pipette controller
- Haemocytometer (cell counting chamber) with Neubauer rulings or Burker counting chamber for cell counts
- Microscope
- Tissue culture flasks, 75 cm2 (or 150 cm2)
- Polyester pressure-sensitive film or microtiter sealing tapes
- pH indicator paper (optional if metabolic activity used as an end-point)

12.1.3 BUFFERS AND REAGENTS

- Minimal Essential Medium (MEM) (commercially available from suitable suppliers)
- Fetal or newborn calf serum (must be confirmed free from diphtheria antitoxin)
- Antibiotic solution containing penicillin (10,000 IU/ml) and streptomycin (10 mg/ml)
- 200 mM L-glutamine solution
- 10% D (+)-Glucose solution
- 1 M HEPES buffer
- Hanks' balanced salt solution (HBSS) or PBS
- 0.25% trypsin/EDTA solution
- Tetrazolium dye 3-(4,5-dimethylthiazol-2-yl)-2,5-diphenyl tetrazolium bromide, MTT
- Sodium lauryl sulfate, SDS
- N,N-dimethylformamide, DMF

12.1.4 COMPLETE CULTURE MEDIUM FOR VERO CELLS

Supplement MEM with:

- calf serum (final concentration 5-10% v/v)
- L-glutamine (2 mM)
- D-glucose (0.1% w/v)
- HEPES (0.01 M)
- penicillin (100 U/ml)
- streptomycin (100 µg/ml)

Other preparations of cell culture medium may also be suitable for use.

- Trypan blue (0.4%) solution

Note: Medium and all the solutions have to be sterile.

If MTT dye is used for staining viable cells, extraction buffer is prepared with solution of sodium lauryl sulfate (SDS, 10% w/v) in dimethylformamide (DMF, 50% v/v, pH to 4.7). Alternatively, other staining reagents may be used (e.g. crystal violet staining solution) prepared with 5 g of crystal violet dissolved in 100 ml of 37% formaldehyde, 200 ml abs. ethanol, 1665 ml distilled water, 35 ml 2 M Tris base and 10 g calcium chloride.

A12.2 PROCEDURES

All procedures are performed aseptically using the laminar airflow cabinet.

12.2.1 CULTURE, HARVESTING AND COUNTING OF VERO CELLS

Established cultures of Vero cells can be maintained in 75 cm^2 tissue culture flasks in complete medium. Depending on the split ratio following passage and the percentage of serum in the medium, a confluent monolayer of cells is obtained after 4-6 days. The following procedure is suitable for routine passage and harvesting of Vero cell cultures. The cells are handled aseptically in the laminar airflow cabinet. Note that one T-75 tissue culture flask of Vero cells at 80-90% confluence will provide enough cells for 3 x 96-well tissue culture plates (at a cell density of 4 x 10^5 cells/ml).

1. Remove the supernatant from a flask containing a confluent monolayer of Vero cells using a sterile pipette.
2. Add 1 ml of sterile HBSS (or PBS) solution to the flask rinse the cells and then remove using a sterile pipette.
3. Add 1 ml of sterile trypsin-EDTA solution to the flask and place in a 37 °C incubator until the cells are detached from the flask (2-5 minutes). The trypsin/EDTA solution should be pre-warmed to 37 °C to speed up the trypsinization process.
4. Add approximately 5 ml of complete medium to the flask to randomize the trypsin and resuspend the cell suspension using a sterile pipette to obtain a suspension of single cells for counting (gently mix the cell suspension within the sterile serological pipette to disperse cell clusters).
5. Prepare a 1 in 5 dilution of the cell suspension in 0.4% trypan blue solution and complete medium (e.g. 100 µl cell suspension + 100 µl 0.4% trypan blue solution + 300 µl complete medium). Depending on the total number of cells present a lower or higher dilution may be required. As a guide, the cell suspension should be diluted such that the total number of cells counted exceeds 100 (minimum required for statistical significance).
6. Prepare the haemocytometer by placing the coverslip over the mirrored counting surface. It may be necessary to moisten the edges of the chamber (this can be done by breathing on the glass) such that Newton's rings (rainbow-like interference patterns) appear indicating that the coverslip is in the correct position to allow accurate cell counting (the depth of the counting chamber is 0.1 mm).
7. Using a pipette, introduce a small sample (approximately 10 µl) of the diluted cell suspension into the counting chamber such that the mirrored surface is just covered. The chamber fills by capillary action. Fill both sides of the chamber to allow for counting in duplicate.
8. The entire grid on a standard haemocytometer is comprised of nine large squares (bounded by 3 lines), each of which has a surface area of 1 mm^2. The total volume of each large square is 1 x 10^{-4} cm^3 (0.0001 ml).
9. Count the number of cells in one large square and calculate the cell concentration as follows: *cells/ml = total cell count in one large square x* 10^4.
10. For example, if 150 cells are counted in one large square (1 mm^2), the concentration of the cell suspension = 150 x 10^4 cells/ml. If fewer than 100 cells are counted in 1 large square it may be necessary to count multiple large squares (for example, the 4 corner squares plus the centre square) and divide the total cell count by the total number of large squares used for counting.
11. For the Vero cell assay, prepare a cell suspension containing approximately 4 x 10^5 cells/ml in complete medium. Note that the cell suspension should be prepared immediately before use and after all dilutions and neutralization steps have been performed.
12. To maintain the culture of Vero cells, seed approximately 1 x 106 cells into a new 75 cm^2 tissue culture flask and add 10 – 15 ml of complete medium prior to incubation at 37 °C.

12.2.2 DETERMINATION OF THE TEST DOSE OF DIPHTHERIA TOXIN

The protocol described here is performed using the Lcd/1000 level of toxin defined by the lowest concentration of toxin (in Lf/ml) which when mixed with 0.001 IU/ml of antitoxin is capable of causing cytotoxic effects on Vero cells after 6 days of culture. At this toxin dose level, the sensitivity of the assay is approximately 0.002 IU/ml for the DI equine antitoxin. The sensitivity of Vero cells to diphtheria toxin may vary when different batches of cells and/or serum are used. As a result, the Lcd/1000 should be determined by each individual laboratory or whenever one of these variables is changed. These parameters should also be confirmed for every new lot of diphtheria toxin and reference antitoxin used. The test dose of toxin is determined by titration of a stable, purified diphtheria toxin against a suitable reference antitoxin as follows:

1. In a sterile 96-well tissue culture plate, fill all the wells of columns 2–11 with 50 µl of complete medium using a multichannel micropipette.
2. Dilute the diphtheria toxin in complete medium to give a starting concentration of approximately 0.02 Lf/ml.

3. Add 100 µl of the diluted diphtheria toxin solution to each well in column 1 using a micropipette.
4. Prepare serial twofold dilutions in 50 µl volumes starting at column 1 through to column 11 using a multichannel micropipette. Discard 50 µl from column 11.
5. Prepare a dilution of the reference diphtheria antitoxin in complete medium to give a diphtheria antitoxin concentration of 0.001 IU/ml.
6. Add 50 µl of the diluted diphtheria antitoxin preparation to all wells in columns 1-11.
7. Add 100 µl of complete medium to 4 "cell control" wells in column 12.
8. Add 50 µl of complete medium and 50 µl of diluted diphtheria antitoxin to 4 "antitoxin control" wells in column 12.
9. Allow the plate to stand at room temperature for 1 hour to allow toxin neutralization to occur.
10. Prepare a suspension of Vero cells in complete medium containing approximately 4 x 105 cells/ml (as described previously).
11. Add 50 µl of the cell suspension to all wells of the microplate. The total volume in all wells is 150 µl.
12. Shake the plates gently and cover with plate sealers to prevent the exchange of gas between medium and air. Note that the use of pressure film to seal plates is an important step for methods based on colour changes in the culture medium to determine assay end-points.
13. Incubate for 6 days at 37 ˚C in 5% CO2 incubator.
14. Perform staining of Vero cells (see example below) or follow an alternative detection method.

The Lcd/1000 dose of diphtheria toxin is defined as the lowest concentration of toxin causing more than 50% cytotoxicity in Vero cells in the presence of 0.001 IU/ml diphtheria antitoxin.

Note that the minimum cytotoxic dose (MCD) of diphtheria toxin can be determined using the same procedure but without the addition of diphtheria antitoxin. To determine the MCD, 50 µl of complete medium should be added in step 6 instead of the diluted diphtheria antitoxin. The antitoxin control wells in column 12 should contain complete medium only and become blank control wells. Because this titration is performed without diphtheria antitoxin, it may be necessary to use a lower starting concentration of diphtheria toxin to determine the MCD.

12.2.3 STAINING OF VERO CELLS

The MTT is reduced in metabolically active, viable cells to form insoluble purple formazan crystals which are then randomized by the addition of detergent or solvent:

1. After 6 days of incubation at 37 ˚C, remove the plate sealer and check the wells for microbial contamination.
2. Prepare a solution of MTT in PBS (5 mg/ml). Sterilize by passing through a 0.2 µM syringe filter. Add 10 µl of the sterile MTT solution to each well of the microplate using a multichannel micropipette.
3. Return the microplate to the 37 ˚C incubator for 4 h to allow metabolism of the MTT by viable cells and formation of the blue formazan product.
4. Carefully remove the medium from all wells using a multichannel micropipette (set to >160 µl).
5. Add 100 µl of extraction buffer to all wells and return the microplate to the 37 ˚C incubator and leave overnight to allow extraction and solubilization of the formazan product.
6. Alternatively, monolayers of Vero cells can be stained for 5 minutes with crystal violet solution dissolved in formalin-ethanol. After staining the solution is discarded and wells rinsed with hot running tap water.

7. The plates are examined visually and the absorbance could be measured at 550-570 nm on a microplate reader. Once staining is complete, the colour is extremely stable at room temperature.

The presence of a dark blue colour indicates viable cells in both methods. A light blue colour indicates partial toxicity, while the absence of colour indicates complete toxicity and cell death.

12.2.4 DETERMINATION OF POTENCY OF THE ANTITOXIN

Serial twofold dilutions of test and reference serum sample are prepared in complete medium in a 96-well tissue culture microplate. After the addition of the test dose of diphtheria toxin (defined in Lcd/1000 and previously determined), the mixtures are incubated at room temperature for 1 h to allow toxin randomization to occur. Vero cells are then added, and the plates incubated for 6 days. After 6 days of culture, the MTT assay or cell staining is performed to determine assay end-points. Reference antitoxin is included on each plate to calculate activity in IU/ml. As an internal control, antitoxin samples of known defined low/high activity may be titrated within each assay.

1. Fill all the wells of columns 2–11 with 50 µl of complete medium using a multichannel micropipette.
2. Fill the first four wells in column 12 (12A–12D) with 100 µl of complete medium using a multichannel micropipette ("cell control").
3. Fill the last four wells in column 12 (12E–12H) with 50 µl of complete medium using a multichannel micropipette ("toxin control").
4. Add 100 µl of each test serum sample into the appropriate well in column 1 (sera from all animals should be randomized across the plates).
5. Prepare a suitable dilution of the reference antitoxin in complete medium (for assays performed at the Lcd/1000 dose level, a starting concentration of 0.064 IU/ml should be suitable). Add 100 µl of the diluted reference antitoxin to the appropriate well in column one in every plate.
6. Make a twofold dilution series in 50 µl volumes starting at column 1 through to column 11 using a multichannel micropipette. Discard 50 µl from column 11 to randomized volumes.
7. Prepare a dilution of the test toxin in complete medium as determined previously. Test dose of toxin is defined as Lcd/1000.
8. Add 50 µl of the diluted diphtheria toxin solution to all wells in columns 1-11 using a multichannel micropipette. Add 50 µl of the diluted diphtheria toxin solution to the last four wells in column 12 (12E – 12H, toxin control).
9. Mix antitoxin with toxin by gently shaking and cover the plate with a lid.
10. Incubate at room temperature (20–25 °C) for one hour to allow toxin neutralization to occur.
11. Meanwhile, prepare a Vero cell suspension in complete medium containing approximately 4 x 105 cells/ml (as described previously).
12. Add 50 µl of the cell suspension to all wells of the microplate. The total volume in all wells should be 150 µl.
13. Shake the plates gently and cover with plate sealers to prevent the exchange of gas between medium and air. Note that the use of pressure film to seal plates is an important step for methods based on colour changes in the culture medium to determine assay end-points.
14. Incubate for 6 days at 37 °C in 5% CO2 incubator.
15. Perform the MTT assay or cell staining as described previously.

A12.3 CALCULATION OF RESULTS

The end-point of each test and reference serum sample is defined as the last well showing neutralization of toxin which in turn can be defined as an optimal density (OD) value greater than the 50% control OD value (if OD recorded). The end-point is recorded as a score based on the dilution of the serum sample at the end-point.

FIGURE A11 Example arrangement of microtiter plate layout for titration of sera using the Vero cells where only one reference sample is included.

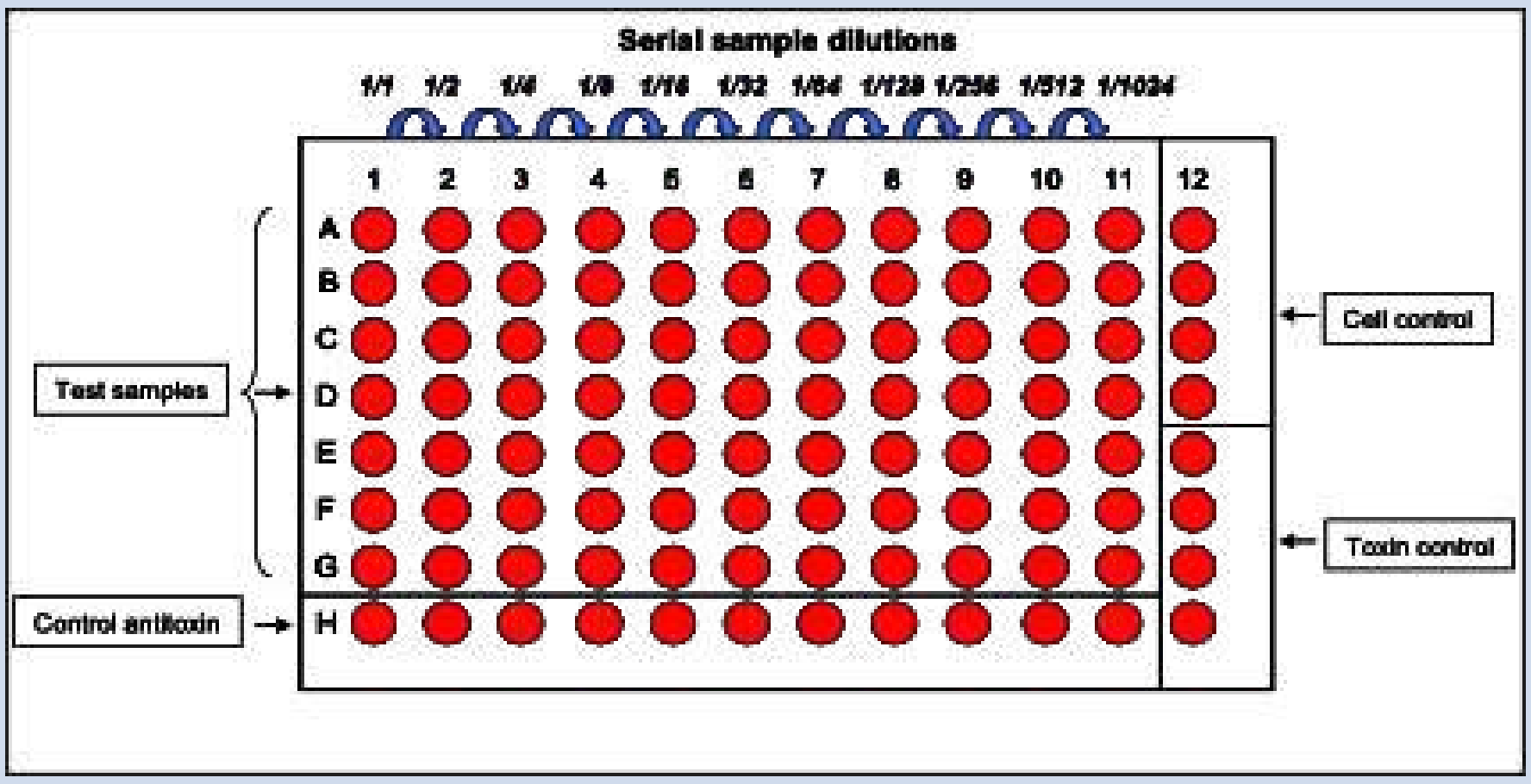

The plate format can be modified to include additional serum controls. The position of the reference (control) antitoxin should be randomized when multiple plates are used.

FIGURE A12 Example of the Vero cell assay following the MTT extraction or staining

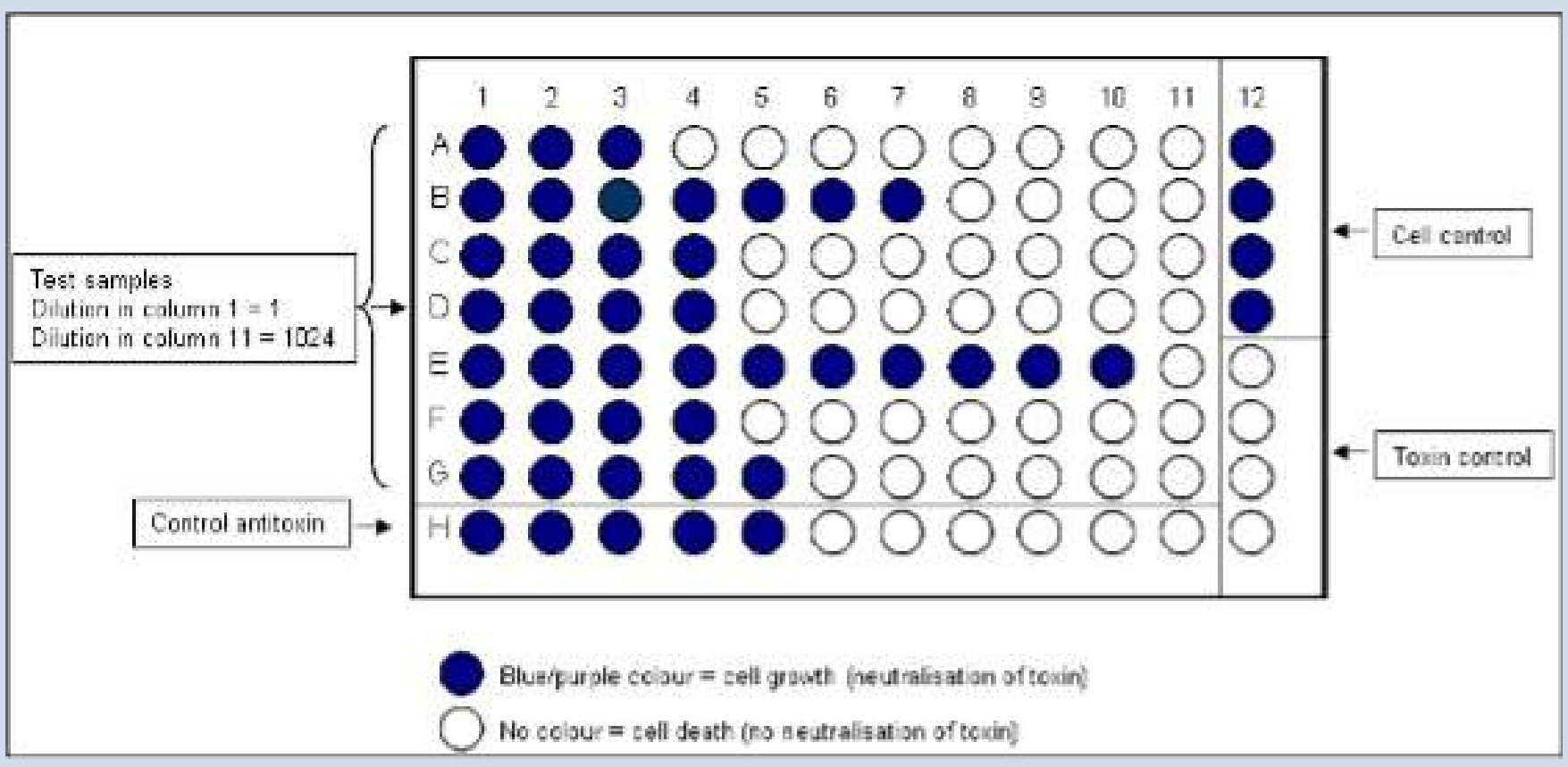

The end-point scores for individual test and reference serum samples should then be converted to titres in IU/ml by comparison with the end-point of the reference antitoxin on each plate. The antibody concentration of each serum under test can be calculated by multiplying the dilution ratio (sample titre end-point or score/standard titre end-point or score) with the calibrated concentration in IU of a reference standard.

Note that any dilution of the serum samples prior to titration in the assay must be taken into account to obtain the final end-point titre in IU/ml.

Note that any dilution of the serum samples prior to titration in the assay must be taken into account to obtain the final end-point titre in IU/ml.

A12.4 VALIDITY OF THE TEST

- The test is not valid if no toxicity is observed in the wells containing Vero cells and diphtheria toxin ("toxin control");
- The test is not valid if the wells containing Vero cells alone (in complete medium, "cell control") do not show positive cell growth with a confluent monolayer of cells after 6 days of culture. As a guide, the OD 570 nm after MTT staining should be >1;
- The end-point for the reference antitoxin should be 0.002 IU/ml for assays performed at the Lcd/1000 dose level;
- The results of the negative control serum must be below the limit of detection and calculated value for the two positive control sera (if used) are within the agreed limits so that the difference between the titres of the lowest and highest standard dilution is not more that 4-fold.

STATISTICAL ANALYSIS PLAN

VERSION 01.00, 12SEP2025

A Phase 1, Randomised, Single-Blind Clinical Study to Evaluate the Safety, Immunogenicity and Tolerability of SPVX02, a Tetanus and Diphtheria Booster Vaccine, Against Two Comparator Vaccines in Healthy Adult Participants

SPL-SPVX02-01

Prepared by: S-cubed Biometrics Ltd

For: Stablepharma Ltd

CONTENTS

1. STATISTICAL ANALYSIS PLAN APPROVAL FORM 4

2. STATISTICAL ANALYSIS PLAN AUTHOR(S) 5

3. LIST OF ABBREVIATIONS 6

4. INTRODUCTION 8

5. STUDY OBJECTIVES 9

6. STUDY DESIGN 10

6.1. Summary of Study Design 10

6.2. Randomisation / Intervention Allocation and Blinding 10

6.3. Blinding 11

6.4. Study Procedures Flow Chart 12

6.5. Schedule of Activities 13

6.6. Interim Analysis / Data Monitoring 15

7. STUDY ENDPOINTS 16

7.1. Primary Safety Endpoints 16

7.2. Secondary Endpoints 16

7.3. Exploratory Endpoints 16

8. SAMPLE SIZE 17

9. STUDY ANALYSIS SETS 18

10. PLANNED STATISTICAL METHODS 19

10.1. Statistical Considerations 19

10.1.1. General Definitions 19

10.1.2. Data Presentation 19

10.1.3. Statistical Testing and Estimation 21

10.1.4. Handling of Dropouts or Missing Data 21

10.1.5. Subgroups 21

10.1.6. Multiple Comparison/Multiplicity 22

10.1.7. Software 22

10.2. Participant Disposition 22

10.3. Protocol Deviations 22

10.4. Demographic and Other Baseline Characteristics 22

10.4.1. Demographics 22

E-Sign ID: dc6e1da8-9522-4e0f-8283-c8e65365bf28

10.4.2. Child-Bearing Potential *22*
10.4.3. Medical History *22*
10.5. Prior and Concomitant Medications 22
10.6. Study Drug Administration 23
10.7. Immunogenicity Analysis 23
10.7.1. Seroprotection *23*
10.7.2. Geometric Mean Antibody Titres *24*
10.7.3. Longer Term Seroprotection *24*
10.8. Safety Analysis 24
10.8.1. Reactogenicity Events *24*
10.8.2. Adverse Events *25*
10.8.3. Laboratory Variables *26*
10.8.4. Pregnancy *27*
10.8.5. Drugs of Abuse Screening *27*
10.8.6. Viral Serology *27*
10.8.7. Vital Signs *27*
10.8.8. Electrocardiogram (ECG) *27*
10.8.9. Physical Examination *27*
11. CHANGES TO THE PROTOCOL SPECIFIED ANALYSIS 28
12. REFERENCES 29
13. TABLES, FIGURES AND LISTINGS 30
13.1. Specific Presentation Details 30
13.2. List of Tables 31
13.3. List of Figures 34
13.4. List of Listings 35
14. TABLE AND LISTING SHELLS 37

1. STATISTICAL ANALYSIS PLAN APPROVAL FORM

	Signature (Date)
**Author(s):**	
Laura Grey<br>Statistics Manager<br>S-cubed Biometrics	Signer Name: Laura Grey<br>Signer Reason: I am the owner of this document<br>Signer Time: 12/Sep/2025 15:14:56<br>9d93d39b-8d10-4db9-a875-18ef874b950d
**Approval(s):**	
Karen O'Hanlon<br>Chief Development Officer<br>Stablepharma Ltd	Karen O'Hanlon<br>Signer Name: Karen O'Hanlon<br>Signer Reason: I approve this document<br>Signer Time: 16/Sep/2025 10:06:26<br>b16b2fe9-43b2-4c6e-b9e2-4b32efd99686
Juana de la Torre Arrieta<br>Development Project Manager<br>Stablepharma Ltd	Juana de la Torre Arrieta<br>Signer Name: Juana De La Torre Arrieta<br>Signer Reason: I approve this document<br>Signer Time: 15/Sep/2025 09:09:41<br>3e51565f-d7bd-49c6-9f3e-f17090b53f05

2. STATISTICAL ANALYSIS PLAN AUTHOR(S)

Laura Grey Msci MSC PhD
Statistics Manager
S-cubed Biometrics Ltd
99 Milton Park
Abingdon
Oxfordshire
OX14 4RY

Email: lag@s-cubed.co.uk

3. LIST OF ABBREVIATIONS

**Abbreviation**	**Definition**
AE	Adverse event
ALP	Alkaline Phosphatase
ALT	Alanine Aminotransferase
BDRM	Blinded Data Review Meeting
BLQ	Below the level of quantification
BMI	Body Mass Index
CI	Confidence Interval
CRF	Case Report Form
CS	Clinically Significant
CSR	Clinical Study Report
ECG	Electrocardiogram
FAS	Full Analysis Set
GM	Geometric Mean
GMT	Geometric Mean Titres
GSD	Geometric Standard Deviation
HCT	Haematocrit
HIV	Human Immunodeficiency Virus
IC	Informed Consent
LLOD	Lower Limit of Detection
LLOQ	Lower Limit of Quantification
LSC	Local Safety Committee
MCV	Mean Corpuscular Volume
MCH	Mean Corpuscular Haemoglobin
MedDRA	Medical dictionary for regulatory activities

Abbreviation	Definition
NCS	Not Clinically Significant
PI	Principal Investigator
PT	Preferred Term
RBC	Red Blood Cells
SAE	Serious adverse event
SAP	Statistical Analysis Plan
SAS	Safety Analysis Set
SD	Standard deviation
SOC	System Organ Class
TE	Treatment emergent
TFL	Tables Figures and Listings
WBC	White Blood Cells
WHO	World Health Organisation
WOCBP	Women of Child-Bearing Potential

4. INTRODUCTION

This statistical analysis plan (SAP) explains in detail the statistical analyses that will be performed for the Stablepharma study SPL-SPVX02-01. The analysis is outlined within the study protocol (version 3.0 dated 22 May 2025), and this SAP contains a more technical and detailed description of those analyses. In particular, information is provided on the definitions of the participant analysis sets, and it also details the list of Tables, Figures and Listings (TFL) that will be produced by S-cubed Biometrics for use and inclusion within the Clinical Study Report (CSR). The SAP has been written and finalised before the database is locked and before provision of any unblinded outputs.

Any deviations from the protocol specified analysis are noted in Section 11. These along with any deviations from the analyses stated within this SAP will be described within the CSR.

5. STUDY OBJECTIVES

The objectives of the study are:

Primary:

- Evaluate the safety of a single dose of SPVX02
- Evaluate the tolerability of a single vaccination of SPVX02, Tetadif® or diTeBooster®

Secondary:

- To evaluate the seroprotection rates observed in sera collected from participants on Day 28 post-dose, following administration of a single dose of SPVX02, Tetadif® or diTeBooster®.
- To evaluate the post vaccination Geometric Mean Titres (GMT) of anti-TT and anti-DT antibodies observed in sera collected from participants at Day 28 post-dose, following administration of a single dose of SPVX02, Tetadif® or diTeBooster®.
- To evaluate the longer-term seroprotection rates observed in sera collected from participants on Day 28 post-dose, following administration of a single dose of SPVX02, Tetadif® or diTeBooster®.

Exploratory:

- To determine the post-vaccination geometric mean titres (GMT) of anti-DT neutralising antibodies observed in sera collected from participants on Day 28 post-dose, following administration of a single dose of SPVX02, Tetadif® or diTeBooster®.

6. STUDY DESIGN

6.1. Summary of Study Design

This is a Phase 1, first-in-human study of SPVX02. This single-blind study will be conducted in 60 healthy adult participants randomised 1:1:1 to receive a single dose of SPVX02, Tetadif® or diTeBooster®. The primary objective is to evaluate safety and tolerability of SPVX02, while the secondary objective includes assessing its immunogenicity. Observations from Tetadif® and diTeBooster® will provide comparative context for these assessments.

The study consists of the following:

- A 42-day screening period before the start of the study intervention and follow up period.
- A study intervention and follow up period of 28 days, consisting of dosing on Day 1, a follow-up telephone call on Day 2, and follow-up visits on Days 7 and 28.
- End of study procedures will be performed at the final visit on Day 28.

In addition, the data from Day 1 to Day 7 (including diary card data) from the first 3 participants (sentinel group) dosed in the study will be reviewed by the Local Safety Committee at Southampton Clinical Research Facility chaired by Prof Chris Edwards BSc, MBBS, MD, FRCP.

The first data review will occur as soon as is reasonably possible after the first 3 participants in the sentinel group have received a single dose of IMP.

This sentinel group of 3 participants will be randomised as follows:

- 2 participants are randomised to receive SPVX02
- 1 participant is randomised to receive Tetadif®
- 0 participants to receive diTeBooster®

The remaining 57 participants in the study will not be dosed until the Local Safety Committee has given its written instruction to proceed.

6.2. Randomisation / Intervention Allocation and Blinding

At the screening visit, participants will be sequentially allocated a participant number (participant ID) upon providing written informed consent. This number will be used to identify the participant throughout the study. The participant number will be 5 digits (SS-NNN) consisting of a two-digit site number (SS) and a three-digit participant number (NNN) (e.g. 001, 002, 003, etc).

The randomisation schedule, generated prior to the study by S-cubed, ensures balanced allocation of participants to the three intervention groups.

- On Day 1, participants will be assigned a unique randomisation number in ascending numerical order. This number encodes the participant's intervention allocation and will be recorded in the study database. The Randomisation number will be a 3 digit number RNN, where R is a one-digit replacement number starting from 0 and NN is a sequential number starting from 01.
- If a participant is withdrawn before receiving the study vaccine, they may be replaced following discussion with the Sponsor. Any replacement participants will be assigned a randomisation number with the next sequential replacement number (R) but the same sequential number (NN). Participants who discontinue after vaccination will not automatically be replaced unless determined necessary by the Sponsor.

- The site will dispense the assigned study intervention at the study visit.
- Study interventions will be labelled with the participant's unique participant number to maintain accurate tracking.
- Once randomisation numbers have been assigned, no attempt will be made to use those numbers again. If a randomisation number is allocated incorrectly, no attempt will be made to remedy the error once the study vaccine has been dispensed.

A sentinel group of 3 participants will be randomised on a ratio of 2:1:0, before the remaining participants are randomised to achieve an overall 1:1:1 ratio.

6.3. Blinding

This is a single-blind study in which participants are blinded to the specific study intervention they receive. The team performing the post-dose assessments, and the sponsor will also remain blinded to intervention allocation. The pharmacy team will be unblinded as will the clinical team performing the study drug administrations. Participants will be randomly assigned in a 1:1:1 ratio to receive SPVX02, Tetadif® or diTeBooster®.

Participants will be randomised on Day 1 prior to dosing where each participant will receive a unique randomisation number. Participants will then receive the corresponding study intervention according to the randomisation scheme. To ensure the number of unblinded personnel is kept to a minimum, the randomisation schedule will only be available to Pharmacy staff. The actual intervention administered should be recorded in the source documentation but not recorded in the eCRF. Blinded personnel will perform key safety and immunogenicity assessments.

6.4. Study Procedures Flow Chart

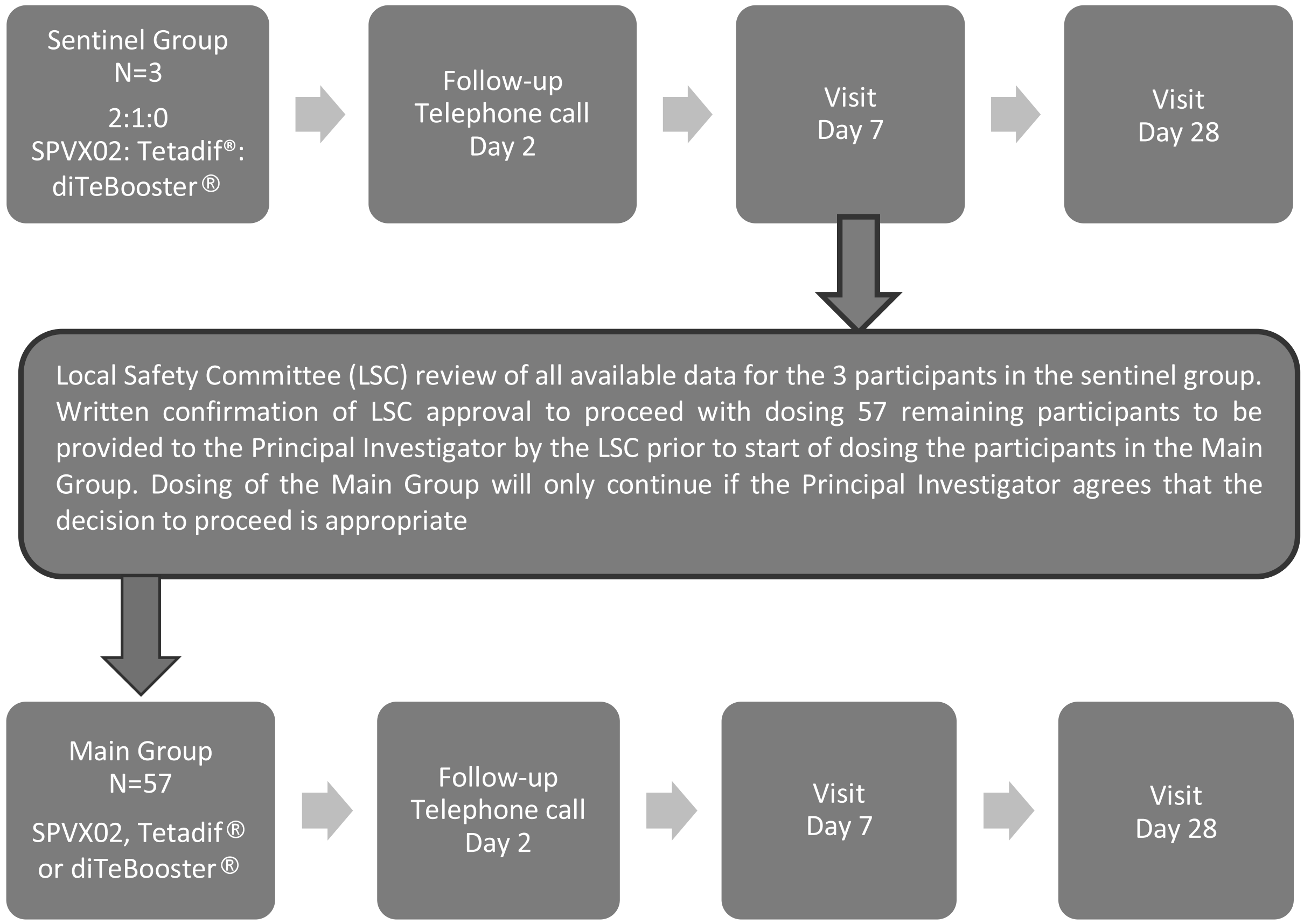

Abbreviations: N= number of participants

Overall, participants will be randomized 1:1:1 for SPVX02:Tetadif®:diTeBooster®

For all participants, all available data relating to safety, immunogenicity, tolerability (including AE and SAE data) will be reviewed on an ongoing basis by the Sponsor's Medical Monitor and the Principal Investigator at the study site. The Sponsor's Medical Monitor and the Principal Investigator will make recommendations concerning the continuation, modification or termination of the study if one of the study stopping criteria defined in Section 9.2 of the protocol is met.. If the study is halted following triggering of a stopping rule, it will only be restarted following approval of a substantial amendment by the regulatory authorities.

In addition, further study oversight will be provided by a designated Local Safety Committee.

6.5. Schedule of Activities

Table 1: Schedule of Activities

**Procedures and Assessments**						
**Study Period**	**Screening**	**Intervention Period**				**ET[j]**
**Visit**	**1**	**2**	**3**	**4**	**5**	
**Day**	**-42 to 0**	**1**	**2**	**7 (± 3 days)**	**28 (± 3 days)**	
Informed consent	X					
Review of eligibility criteria by the PI[b]	X	X[c]				
Demographics and baseline characteristics	X					
Full physical examination[d]	X	X		X[h]	X[h]	
Height and weight	X					
Medical history	X					
Clinical laboratory tests (biochemistry, haematology, coagulation, thyroid function)	X	X			X	X
Urinalysis	X	X			X	X
Pregnancy test (WOCBP only)	X	X				
Drug and alcohol screen	X	X				
HIV, Hepatitis B and C screen	X					
12-lead ECG[e]	X[f]					
Vital signs	X	X		X[h]	X[h]	
Randomisation		X				
Dosing		X				
AE review[g]	X	X	X	X	X	X
Concomitant medications	X	X	X	X	X	X
Blood samples for tetanus and diphtheria antibody analysis	X	X			X	X
Blood samples for microneutralisation assay		X			X	
Local and systemic reactogenicity daily evaluation (pain, redness, swelling, etc.) – e-diary (for first 7 days post dose only)		X[i]	X[i]	X[i]		
Telephone call to assess concomitant medications and AEs			X			

[a] In the case of a participant withdrawing from a study (between intervention and follow up (Day 1 to Day 28)), or being withdrawn by the investigator, a written confirmation of the withdrawn will be provided and signed by the investigator. Criteria for withdrawal of participants from the study are described in the protocol.

[b] Eligibility must be confirmed by a qualified medical doctor who has received relevant training in this study and is entered on the study delegation log

[c] Eligibility must be re-confirmed at randomisation prior to first dose of investigational intervention

E-Sign ID: dc6e1da8-9522-4e0f-8283-c8e65365bf28

[d] Full physical examination.
[e] 12-lead ECGs will be obtained.
[f] At screening, a 12-lead ECGs will be obtained
[g] AE and concomitant medication review will be carried out throughout the study from informed consent to EoS.
[h] To be symptom directed and performed at the discretion of the Investigator
[i] e-Diaries are recorded by participants each day (day 1 to 7)
[j] Further details for the Early Termination are described in the protocol.

AE=adverse event; ECG=electrocardiogram; ET=early termination; HIV=human immunodeficiency virus; EoS=end of study; PI=principal investigator; WOCBP=women of childbearing potential.

6.6. Interim Analysis / Data Monitoring

There will be no interim analysis for this study. The safety of the sentinel group will be assessed prior to proceeding with the remainder of the study. Additionally a Local Safety Committee (LSC) may monitor the data for the study. This will be done in a blinded manner unless unblinding is necessary, in which case the relevant outputs will be restricted to a small unblinded team.

7. STUDY ENDPOINTS

7.1. Primary Safety Endpoints

Incidence of safety and reactogenicity events which include:

- Adverse events
- Serious adverse events
  - Incidence of local and systemic reactogenicity events observed for 7 days post-dose which include: pain, induration/swelling, tenderness, warmth, erythema at the vaccination site plus feverishness, chills, myalgia (muscle ache) , fatigue, headache, arthralgia (joint pain), (skin) rash.

7.2. Secondary Endpoints

Secondary endpoints will include:

- Incidence of seroprotection resulting from a single dose of SPVX02, Tetadif® or diTeBooster® at 28 days post-dose.
- Evaluation of GMT of anti-TT and anti-DT antibodies resulting from a single dose of SPVX02, Tetadif® or diTeBooster® at 28 days post-dose.
- Incidence of longer term seroprotection resulting from a single dose of SPVX02, Tetadif® or diTeBooster® at 28 days post-dose.

7.3. Exploratory Endpoints

- Determination of geometric mean neutralising antibody titres (GMT) for diphtheria resulting from a single vaccination with SPVX02, Tetadif® or diTeBooster® at 28 days post-dose.

Exploratory endpoints will not be described within this SAP.

8. SAMPLE SIZE

The protocol specified sample size section is as follows:

As there is no formal hypothesis testing for this study there has been no formal sample size estimation. However, 20 participants in each of the 3 intervention groups is judged to be sufficient so as to meet the study objectives.

9. STUDY ANALYSIS SETS

Analysis sets defined below will be reviewed against the study database at a blinded data review meeting (BDRM). The database at this time will be nearly final (i.e. meeting may result in further data queries/changes post meeting), so participant inclusion/exclusion from analysis sets defined at this meeting, will be further checked (post meeting) against a locked database, and will then be finalised prior to unblinding the study.

**Analysis Sets**	**Description**
Full Analysis Set (FAS)	All randomised participants.
Safety Analysis Set (SAS)	All randomised participants who were exposed to an investigational intervention.
Immunogenicity Analysis Set	All randomised participants who were exposed to an investigational intervention and have at least one baseline and one corresponding post-baseline immunogenicity measurement and have no protocol deviations that may impact their immunogenicity results.

10. PLANNED STATISTICAL METHODS

10.1. Statistical Considerations

10.1.1. General Definitions

In all applicable summary/analysis presentations of safety endpoints, Baseline is defined as the last non-missing assessment value for a participant, for that particular parameter, that is prior to dosing with study intervention, unless over-ruled at the BDRM or otherwise stated in the appropriate endpoint sections below.

Within summary presentations/analyses it is envisaged that only scheduled protocol visit values will be used for post-baseline time points. In the clinical database a number of data points have been labelled as unscheduled/additional recordings of data. These data points will be included within participant listings only. However, at the BDRM the occurrence of such unscheduled data will be reviewed to decide if (and how) any such data points should be included within summary presentations/analyses. Any such decisions will be documented in the BDRM minutes.

10.1.2. Data Presentation

The full list of TFLs to be produced for the final study analysis are shown in Section 13, and the specific format and content of each data Table/Listing presentation is shown in Section 14.

All summaries will be presented by intervention group. Selected presentations will include an overall column which will include all three intervention groups.

Within all Tables and Figures the interventions will be labelled as in Table 2, while the abbreviated labels may be used in the listings if required. Listings will be presented by intervention group and participant number.

Table 2: Intervention Group Labels

Label	Abbreviated Labels
SPVX02	SPVX
Tetadif	Teta
diTeBooster	diTe

The scheduled protocol visits will be labelled in tables and figures as follows:

- Baseline
- Day 1
- Day 7
- Day 28

The baseline definition may vary by endpoint, as stated in Section 10.1.1 and the specific endpoint sections. Day 1 is the day that the intervention is administered following baseline safety measurements.

An electronic reactogenicity diary is completed every day following intervention from Day 1 to Day 7 (inclusive), the visits labels for the data collected within the diary will be presented in relevant summary tables as Day 1, Day 2, Day 3, Day 4, Day 5, Day 6 and Day 7.

Listings will include all relevant data collected within the study database, with the visit listed as recorded.

Unscheduled visit data will be labelled as “Unscheduled” together with a date in the relevant data Listings. Unscheduled data will not be used in the summary tables unless otherwise agreed at the BDRM.

Where duplicate information is collected in both the database and in the vendor data transfer(s) (e.g., sampling date and time) this information will be reconciled by data management and the information from the database will be included in participant Listings.

All variables will be listed to the same number of decimal places as reported. Descriptive statistics for all endpoints that are continuous data will have the following summary statistics presented in the following order: n, mean (rounded to one more decimal place than recorded), standard deviation (rounded to two more decimal places than recorded), median (rounded to one more decimal place than recorded), lower quartile (rounded to one more decimal place than recorded), upper quartile (rounded to one more decimal place than recorded), minimum (as recorded), and maximum (as recorded).

Note: for endpoint(s) that require a geometric mean to be produced, and those endpoint(s) can have raw values of 0 (zero), the geometric mean calculation will add an appropriate constant value to all raw values prior to logging and will subtract that constant value from the final calculated anti-logged mean. The constant value used will be documented in the footnote of the tables. An example of such a calculation is shown below:

$$GM = \left(10^{\left[\frac{\sum_{i=1}^{N} log_{10}(x_i+1)}{N}\right]} \right) - 1$$

Categorical variables will be summarised using proportions (counts and percentages). All percentages in summary tables will be calculated using as the denominator, either the participant analysis set or the number of non-missing observations. The specific approach is detailed within each (relevant) table template (Section 14).

Unless otherwise stated in the appropriate endpoint section(s) below, laboratory parameter values that are <X, below the level of quantification (BLQ), or less than the lower limit of quantification

(<LLOQ) will be set to zero in computations for summary presentations and analysis but will be noted as collected in Listings.

10.1.3. Statistical Testing and Estimation

There will be no statistical testing in this study. Where appropriate 95% confidence intervals may be presented.

10.1.4. Handling of Dropouts or Missing Data

In general, missing data will not be imputed and all summary statistics will be reported based upon observed data. For a limited number of summary presentations, missing data rules may be introduced. In particular, methods of handling incomplete dates for adverse events (Section 10.9.1) and incomplete dates for concomitant medications (Section 10.5) are presented. Any other data with partial dates, which are required for use in time related calculations, will be completed using a suitably conservative approach. Dates will be shown in Listings as they have been recorded.

10.1.5. Subgroups

For both diphtheria and tetanus the seroprotection status at baseline will be derived for each participant. A participant was seroprotected at baseline if their IgG serum antibody titre was ≥ 0.1 IU/mL.

Summaries may be presented by seroprotection status at baseline, or for only the subgroup of participants who were not seroprotected at baseline. Similarly summaries may be presented by whether a participant had achieved longer-term seroprotection at baseline or not, where this is derived for both diphtheria and tetanus if the IgG serum antibody was > 1.0 IU/mL.

Other subgroups that may be used include:

- Sex
    - Male
    - Female
- BMI Group
    - <18.5 – Underweight
    - 18.5 – 24.9: Normal Weight
    - 25.0 – 29.9: Overweight
    - 30.0 – 34.9: Obese Class I
    - 35.0 – 39.9: Obese Class II
    - >= 40.0: Obese Class III
- Age Group
    - <=25
    - 26-35
    - 36-45
    - >45

Age groups or BMI groups with less than 2 participants over the three intervention groups may be merged.

10.1.6. Multiple Comparison/Multiplicity

As there is no statistical testing no adjustment for multiple comparisons is required.

10.1.7. Software

Data will be reported using SAS (version 9.4 or later).

10.2. Participant Disposition

The number of participants enrolled (non-screen failure), the number randomised, the number withdrawing (also split by reason for withdrawal) from and completing the study, and the numbers in each analysis set will be summarised by intervention group. The data on participant disposition, informed consent and analysis set membership will also be listed.

10.3. Protocol Deviations

Protocol deviations will be listed using information collected on the CRF and will be described appropriately within the CSR. Important protocol deviations (i.e. those that may impact the analysis) will be discussed at the BDRM.

10.4. Demographic and Other Baseline Characteristics

Unless otherwise described, the Safety Analysis Set will be used in summaries of demographic and baseline data. No statistical testing will be used to compare interventions for different baseline characteristics.

10.4.1. Demographics

Demographic variables at informed consent (sex, age, race, ethnicity, height (cm), weight (kg), body mass index (kg/m^2) and child-bearing potential) will be summarised across all participants.

10.4.2. Child-Bearing Potential

Child-bearing potential and contraceptive use will be listed.

10.4.3. Medical History

Past and present significant medical history data will be coded using MedDRA version 28.0. Ongoing medical history, identified as those conditions of medical history that were ongoing at screening, will be summarised by MedDRA system organ class (SOC) and preferred term by intervention group.

10.5. Prior and Concomitant Medications

All medication terms will be coded using the World Health Organisation (WHO) Drug Dictionary Enhanced (WHO Drug Global, version March 2025).

Medications will be assigned as being prior to or concomitant with study intervention, based on the start and stop dates of the medication and the date of study intervention. If the medication stop date is before the date of study intervention, the medication will be assigned as being prior to study intervention. Medications that are ongoing at administration of study intervention or started after

first administration will be deemed to be concomitant medications. If medication dates are incomplete and it is not clear whether the medication was concomitant, it will be assumed to be concomitant.

All concomitant medications will be summarised by ATC class (Level 2) and preferred base name (WHO Code 01001) by intervention group. If a participant has separate periods of taking specific medications, then that medication is only counted once within the specific period of observation (i.e. prior or concomitant) where it is taken. All medications will be listed, where concomitant medications will be flagged.

10.6. Study Drug Administration

Details of study intervention administration, including date, time and intervention received will be listed.

10.7. Immunogenicity Analysis

All immunogenicity analyses will be performed on the Immunogenicity Analysis Set and participants will be summarised/analysed by the actual intervention.

Any antibody results that are below the lower limit of quantification (LLOQ) will be reported as measured, while the results below the lower limit of detection (LLOD) will be reported as <LLOD. Any results <LLOD will be replaced with half of the LLOD before summarising. Values above the LLOD will be summarised as reported.

All antibody results will be listed as reported, with any relevant limits (LLOD and/or LLOQ) highlighted.

10.7.1. Seroprotection

Seroprotection is defined as an IgG serum antibody titre of ≥ 0.1 IU/mL which is the antibody titre level of anti-TT and anti-DT antibodies that are considered to confer protection against tetanus[1] and diphtheria [2] infection.

The seroprotection rate for tetanus and diphtheria will be summarised by intervention group as the number and percentage of participants who meet the criteria for seroprotection at Day 28. The summary will also present the 95% Clopper-Pearson Confidence interval for each group.

Seroprotection rate will be presented similarly for the subgroup of participants who were not seroprotected at baseline, and who were not longer-term seroprotected at baseline.

To account for the possibility of participants already meeting the protocol defined seroprotection criteria at baseline additional definitions of seroprotection have been included to allow further comparison of the study interventions.

1) IgG serum antibody titre of >= 0.1 IU/mL and an increase from baseline of at least 2-fold.
2) IgG serum antibody titre of >= 0.1 IU/mL and an increase from baseline of at least 4-fold.

For both of these definitions the seroprotection rate for tetanus and diphtheria will be summarised as described above.

10.7.2. Geometric Mean Antibody Titres

The absolute antibody titres will be summarised, at both baseline and Day 28, by intervention group. The summary will include the geometric mean and standard deviation.

The summary will also include the geometric mean ratio of the Day 28 titre to the baseline titre. This will be derived by back-transforming the mean of the [log(titre at Day 28) – log(titre at Baseline)].

A box-scatterplot will be produced of the antibody titres, separately for tetanus and diphtheria. Each page will present the titres at baseline and at Day 28 for each of the 3 intervention groups. The y-axis will be presented on the log-scale.

Additionally summary tables and box-scatterplots will be presented by seroprotection status at baseline, longer-term seroprotection status at baseline, age group, sex and BMI group.

10.7.3. Longer Term Seroprotection

Longer-term seroprotection is defined as an IgG serum antibody titre of >= 1.0 IU/mL which is the antibody titre level of anti-TT and anti-DT antibodies that are considered to confer longer-term protection against tetanus[1] and diphtheria [2] infection.

The longer-term seroprotection rate for tetanus and diphtheria will be summarised by intervention group as the number and percentage of participants who meet the criteria at Day 28. The summary will also present the 95% Clopper-Pearson Confidence interval for each group.

Longer-term seroprotection rate will be described similarly for the subgroup of participants who were not seroprotected at baseline and those who were not longer-term seroprotected at baseline.

Additional definitions have also been added for longer-term seroprotection:

1) IgG serum antibody titre of >=1.0 IU/mL and an increase from baseline of at least 2-fold.
2) IgG serum antibody titre of >=1.0 IU/mL and an increase from baseline of at least 4-fold.

For both of these definitions the seroprotection rate for tetanus and diphtheria will be summarised as described above.

10.8. Safety Analysis

The SAS will be used for all safety presentations. All analyses of safety endpoints will be descriptive and no statistical analysis of safety data will be performed. Any presentations of safety data by intervention group will be presented by actual intervention.

10.8.1. Reactogenicity Events

An e-diary will be completed by participants every evening from Day 1 to Day 7 to assess the tolerability of the study interventions. The diary will collect information on symptoms including temperature, reactions local to the injection site (pain, induration/swelling, tenderness, warmth, erythema/redness) and systemic reactions (feverishness, chills, muscle ache, fatigue, headache, joint pain, skin rash).

Where a participant has reported a symptom (i.e. answered yes to having experienced the symptom) a grade will be derived using the information from the e-diary using Table 3. Any temperature below 38.0 ^{O}C will be classified as not having a fever.

Table 3: Severity Grading for Reactogenicity Events

	Mild (Grade 1)	Moderate (Grade 2)	Severe (Grade 3)	Potentially Life Threatening (Grade 4)
Fever (^{O}C)	38.0-38.4	38.5-38.9	39.0-40.0	>40.0
Erythema/Redness (cm)	2.5-5.0	5.1-10.0	>10.0	Necrosis or exfoliative dermatitis
Induration/Swelling (cm)	2.5 - 5.0 and no interference	5.1 - 10.0 or some interference	>10.0 or significant interference	Necrosis
Pain	no interference	Repeated use of pain relief > 24 hour or some interference	significant interference	ER visit or hospitalisation
Warmth	no interference	some interference	significant interference	ER visit or hospitalisation
Tenderness	Mild discomfort to touch	Discomfort with movement	Significant discomfort at rest	ER visit or hospitalisation
Feverishness	no interference	some interference	significant interference	ER visit or hospitalisation
Chills	no interference	some interference	significant interference	ER visit or hospitalisation
Muscle Ache	no interference	some interference	significant interference	ER visit or hospitalisation
Fatigue	no interference	some interference	significant interference	ER visit or hospitalisation
Headache	no interference	Repeated use of pain relief > 24 hour or some interference	significant interference	ER visit or hospitalisation
Joint Pain	no interference	some interference	significant interference	ER visit or hospitalisation
Skin Rash	no interference	some interference	significant interference	ER visit or hospitalisation

The number and percentage of participants with each symptom, or any symptom, will be summarised by intervention group and study day, as well as the number and percentage of participants who experience the symptom at any time over the 7 days. Additionally the summary will be presented by severity. In the summary by severity the 'any time' and 'any symptom' summary will present the worst severity of the symptoms.

10.8.2. Adverse Events

All unsolicited adverse events will be coded using MedDRA, Version 28.0. This may include adverse events identified through the symptom e-diaries.

An adverse event is defined as treatment-emergent if the onset date is on or after the date of first dose of study intervention. Should any onset date for an adverse event be missing or only a partial date recorded (such that it cannot be determined if the event onset was prior to the date of first dose) then it will be assumed that the event is treatment-emergent, unless the adverse event stop date indicates otherwise. Any adverse event with an onset date earlier than the date of first dose of study

intervention will be classified as a pre-treatment adverse event and will be identified in participant listings only.

If a participant experiences more than one AE with the same preferred term, that preferred term will be counted only once. It will be assigned the greatest observed severity and the strongest relationship to study intervention among those events for the tables in which those characteristics are summarised.

Treatment-emergent AEs (participant incidence and number of events) will be summarised by:

- Intervention group, system organ class, and preferred term;
- Intervention group, system organ class, preferred term and severity;

Additionally serious treatment-emergent AEs (participant incidence) will be summarised by intervention group, system organ class, and preferred term, as well as related treatment-emergent adverse events where related includes any AE that has been recorded as possible, probable, unlikely, unknown or the relationship is missing.

In all AE summary tables results will be displayed ordered in terms of decreasing frequency of SOC occurrence (based on the total across intervention groups), and within each SOC also ordered in terms of decreasing frequency of preferred term occurrence (also based on the total across intervention groups). In all AE summary tables, in addition to presenting the information by intervention group, a total column (all three intervention groups combined) will also be presented.

10.8.3. Laboratory Variables

The following haematology, biochemistry, coagulation and thyroid parameters will be included within summary presentations (and presented in the units as shown):

- Haematology: Red blood cell (RBC) count (10^12/L), mean corpuscular haemoglobin (MCH) (pg), mean corpuscular volume (MCV) (fL), haematocrit (HCT) (%), haemoglobin (g/L), platelet count (10^9/L), white blood cell (WBC) count (absolute, 10^9/L), neutrophils (absolute, 10^9/L), lymphocytes (absolute, 10^9/L), monocytes (absolute, 10^9/L), eosinophils (absolute, 10^9/L), basophils (absolute, 10^9/L).

- Coagulation: Prothrombin time (s), Prothrombin Intl. normalised ratio (ratio), activated partial thromboplastin time (s).

- Thyroid Function: Thyroid stimulating hormone, Thyroxine, free.

- Biochemistry: Alanine aminotransferase (ALT) (IU/L), albumin (g/L), alkaline phosphatase (ALP) (IU/L), total bilirubin (umol/L), calcium (mmol/L), creatinine (umol/L), glucose (mmol/L), potassium (mmol/L), total protein (g/L), sodium (mmol/L), urea (mmol/L).

Laboratory data collected in different units to that shown will be converted to the above specified units (if possible) for presentation in tables and listings.

Laboratory values outside the reference range will be identified in the participant listings as above or below the reference range. Laboratory values that are below the level of quantification (BLQ) or <X will be set to zero in computations for summary presentations but will be noted as recorded (i.e. BLQ or <X) in participant listings. Laboratory values that are missing will remain missing and will not be presented in listings.

Observed parameter values and changes from baseline will be summarised for the above highlighted haematology, biochemistry, coagulation and thyroid parameters by intervention group at each protocol scheduled visit.

Unscheduled visit assessments will be included within the relevant participant listings.

Urinalysis results and, where available, microscopy results will be listed.

10.8.4. Pregnancy

The results of pregnancy tests, both urine and serum, carried out throughout the study will be listed for all females.

10.8.5. Drugs of Abuse Screening

The results of drugs of abuse screening will be listed.

10.8.6. Viral Serology

The results of viral serology screening (HBsAg, HCV and HIV) will be listed.

10.8.7. Vital Signs

All vital signs results will be listed.

10.8.8. Electrocardiogram (ECG)

The ECG results obtained at screening will be listed.

10.8.9. Physical Examination

Physical examination results will be listed.

11. CHANGES TO THE PROTOCOL SPECIFIED ANALYSIS

The following changes have been made in the SAP compared to the protocol specified analysis:

Additional seroprotection definitions have been included to account for the possibility of participants having a high antibody level prior to receiving the study vaccination.

Two subgroups, based on the baseline antibody levels (seroprotection and longer-term seroprotection) have also been added along with a number of presentations that will be displayed by these subgroups.

Additionally a number of presentations will be displayed by Age group, BMI group and Sex.

12. REFERENCES

1 World Health Organization (2017) Weekly Epidemiological Record. Vol. 92, World Health Organization, Geneva, 53-76.

2 World Health Organization (2017) Weekly Epidemiological Record. Vol. 92, World Health Organization, Geneva, 417-436.

13. TABLES, FIGURES AND LISTINGS

13.1. Specific Presentation Details

Tables, listings and figures will be provided in word and pdf format. All summary tables and figures will have source data footnotes that refer to the relevant listings. Dates will appear as DDMMMYYYY times as HH:MM. All listings will be ordered by Intervention group, participant number and date/time of visit. For the presentation of summary data, values will be aligned based on the unit column, and not left/right justified. For example:

```
 Parameter                         n                XX
                                   Mean (SD)        XX.X (XX.XX)
                  [optional]       GM (GSD)         XX.X (XX.XX)
                  [optional]       CV (%)           XX.X
                                   Median           XX.X
                                   Q1, Q3           XX.X, XX.X
                                   Min, Max         XX, XX
```

All Tables, Listings and Figures will have the SAS program name and date of production in the footnote.

All Tables, Listings and Figures will include the following study header and footer:

```
CONFIDENTIAL                         Stablepharma                Page x of y
                                    SPL-SPVX02-01

                                      Table X.X
                                        Title
                                     Analysis Set









Source Data: Listing 16.2.x [Source data footnote only appears for tables and figures, where x references
relevant listing number]

Program: XXXXXXXXX                 Output: XXXXXXX             Date: XXXXXXXXX
```

13.2. List of Tables

Table Number	Table Title
14.1.1	Participant Disposition – All Participants
14.1.2	Demography – Safety Analysis Set
14.1.3	Medical History by System Organ Class and Preferred Term – Safety Analysis Set
14.1.4	Concomitant Medications – Safety Analysis Set
14.2.1.1.1	Seroprotection Rate – Diphtheria – Immunogenicity Analysis Set
14.2.1.1.2	Seroprotection Rate – Diphtheria - Participants Not Seroprotected at Baseline – Immunogenicity Analysis Set
14.2.1.1.3	Seroprotection Rate – Diphtheria - Participants Not Longer-Term Seroprotected at Baseline – Immunogenicity Analysis Set
14.2.1.2.1	Seroprotection Rate – Tetanus – Immunogenicity Analysis Set
14.2.1.2.2	Seroprotection Rate – Tetanus - Participants Not Seroprotected at Baseline – Immunogenicity Analysis Set
14.2.1.2.3	Seroprotection Rate – Tetanus - Participants Not Longer-Term Seroprotected at Baseline – Immunogenicity Analysis Set
14.2.2.1.1	Geometric Mean Antibodies - Diphtheria – Immunogenicity Analysis Set
14.2.2.1.2	Geometric Mean Antibodies - Diphtheria – By Seroprotection Status at Baseline – Immunogenicity Analysis Set
14.2.2.1.3	Geometric Mean Antibodies - Diphtheria – By Longer-Term Seroprotection Status at Baseline – Immunogenicity Analysis Set
14.2.2.1.4	Geometric Mean Antibodies - Diphtheria – By Age Group – Immunogenicity Analysis Set
14.2.2.1.5	Geometric Mean Antibodies - Diphtheria – By Sex – Immunogenicity Analysis Set
14.2.2.1.6	Geometric Mean Antibodies - Diphtheria – By BMI Group – Immunogenicity Analysis Set
14.2.2.2.1	Geometric Mean Antibodies - Tetanus – Immunogenicity Analysis Set
14.2.2.2.2	Geometric Mean Antibodies - Tetanus – By Seroprotection Status at Baseline – Immunogenicity Analysis Set

Table Number	Table Title
14.2.2.2.3	Geometric Mean Antibodies - Tetanus – By Longer-Term Seroprotection Status at Baseline – Immunogenicity Analysis Set
14.2.2.2.4	Geometric Mean Antibodies - Tetanus – By Age Group – Immunogenicity Analysis Set
14.2.2.2.5	Geometric Mean Antibodies - Tetanus – By Sex– Immunogenicity Analysis Set
14.2.2.2.6	Geometric Mean Antibodies - Tetanus – By BMI Group – Immunogenicity Analysis Set
14.2.3.1.1	Longer-Term Seroprotection Rate – Diphtheria – Immunogenicity Analysis Set
14.2.3.1.2	Longer-Term Seroprotection Rate – Diphtheria - Participants Not Seroprotected at Baseline – Immunogenicity Analysis Set
14.2.3.1.3	Longer-Term Seroprotection Rate – Diphtheria - Participants Not Longer-Term Seroprotected at Baseline – Immunogenicity Analysis Set
14.2.3.2.1	Longer-Term Seroprotection Rate – Tetanus – Immunogenicity Analysis Set
14.2.3.2.2	Longer-Term Seroprotection Rate – Tetanus - Participants Not Seroprotected at Baseline – Immunogenicity Analysis Set
14.2.3.2.3	Longer-Term Seroprotection Rate – Tetanus - Participants Not Longer-Term Seroprotected at Baseline – Immunogenicity Analysis Set
14.3.1.1	Reactogenicity Events Summary – Safety Analysis Set
14.3.1.2	Reactogenicity Events Summary by Severity – Safety Analysis Set
14.3.2.1	Overall Summary of Treatment-Emergent Adverse Events – Safety Analysis Set
14.3.2.2	Treatment-Emergent Adverse Events by System Organ Class and Preferred Term – Safety Analysis Set
14.3.2.3	Treatment-Emergent Adverse Events by System Organ Class, Preferred Term and Severity – Safety Analysis Set
14.3.2.4	Treatment-Emergent Adverse Events by System Organ Class, Preferred Term and Relationship to Study Intervention – Safety Analysis Set
14.3.2.5	Serious Treatment-Emergent Adverse Events by System Organ Class and Preferred Term – Safety Analysis Set

Table Number	Table Title
14.3.2.6	Treatment-Emergent Adverse Events Leading to Death or Discontinuation by System Organ Class and Preferred Term – Safety Analysis Set
14.3.3.1	Listing of Participants with Serious Adverse Events – Safety Analysis Set
14.3.3.2	Listing of Participants with Adverse Events Directly Resulting in Withdrawal – Safety Analysis Set
14.3.4.1	Haematology – Change from Baseline Summary – Safety Analysis Set
14.3.4.2	Chemistry – Change from Baseline Summary – Safety Analysis Set
14.3.4.3	Coagulation – Change from Baseline Summary – Safety Analysis Set
14.3.4.4	Thyroid Function – Change from Baseline Summary – Safety Analysis Set

13.3. List of Figures

Figure Number	Figure Title
14.2.1.1.1	Box-Scatter Plot of Diphtheria Antibodies Titre – Immunogenicity Analysis Set
14.2.1.1.2	Bar Chart of Diphtheria Antibodies Titre – Immunogenicity Analysis Set
14.2.1.2.1	Box-Scatter Plot of Diphtheria Antibodies Titre - By Seroprotection Status at Baseline – Immunogenicity Analysis Set
14.2.1.2.2	Bar Chart of Diphtheria Antibodies Titre - By Seroprotection Status at Baseline – Immunogenicity Analysis Set
14.2.1.3.1	Box-Scatter Plot of Diphtheria Antibodies Titre - By Longer-Term Seroprotection Status at Baseline – Immunogenicity Analysis Set
14.2.1.3.2	Bar Chart of Diphtheria Antibodies Titre - By Longer-Term Seroprotection Status at Baseline – Immunogenicity Analysis Set
14.2.1.4	Box-Scatter Plot of Diphtheria Antibodies Titre - By Age Group – Immunogenicity Analysis Set
14.2.1.5	Box-Scatter Plot of Diphtheria Antibodies Titre - By Sex – Immunogenicity Analysis Set
14.2.1.6	Box-Scatter Plot of Diphtheria Antibodies Titre - By BMI Group – Immunogenicity Analysis Set
14.2.2.1.1	Box-Scatter Plot of Diphtheria Antibodies Titre – Immunogenicity Analysis Set
14.2.2.1.2	Bar Chart of Tetanus Antibodies Titre – Immunogenicity Analysis Set
14.2.2.2.1	Box-Scatter Plot of Tetanus Antibodies Titre - By Seroprotection Status at Baseline – Immunogenicity Analysis Set
14.2.2.2.2	Bar Chart of Tetanus Antibodies Titre - By Seroprotection Status at Baseline – Immunogenicity Analysis Set
14.2.2.3.1	Box-Scatter Plot of Tetanus Antibodies Titre - By Longer-Term Seroprotection Status at Baseline – Immunogenicity Analysis Set
14.2.2.3.2	Bar Chart of Tetanus Antibodies Titre - By Longer-Term Seroprotection Status at Baseline – Immunogenicity Analysis Set
14.2.2.4	Box-Scatter Plot of Tetanus Antibodies Titre - By Age Group – Immunogenicity Analysis Set
14.2.2.5	Box-Scatter Plot of Tetanus Antibodies Titre - By Sex – Immunogenicity Analysis Set
14.2.2.6	Box-Scatter Plot of Tetanus Antibodies Titre - By BMI Group – Immunogenicity Analysis Set

13.4. List of Listings

Listing Number	Listing Title
16.2.1.1	Participant Disposition
16.2.1.2	Failed Inclusion and Exclusion Criteria
16.2.2	Protocol Deviations
16.2.3	Participant Analysis Sets
16.2.4.1	Demographic Characteristics and Body Measurements
16.2.4.2	Child-Bearing Potential and Contraception Use
16.2.4.3	Medical History
16.2.4.4	Prior and Concomitant Medications
16.2.4.5	Substance Use History
16.2.5.1	Study Drug Administration
16.2.5.2	Post-Administration Observation
16.2.6.1	Antibody Titres
16.2.6.2	Seroprotection
16.2.7.1	Reactogenicity Events
16.2.7.2	Adverse Events
16.2.8.1	Haematology
16.2.8.2	Chemistry
16.2.8.3	Coagulation
16.2.8.4	Thyroid Function
16.2.8.5	Urinalysis
16.2.9.1	Pregnancy Tests
16.2.9.2	Drugs of Abuse Screen
16.2.9.3	Viral Serology
16.2.10	Vital Signs
16.2.11	Electrocardiogram

Listing Number	Listing Title
16.2.12	Physical Examination
16.2.13	Visit Dates

14. TABLE AND LISTING SHELLS

E-Sign ID: dc6e1da8-9522-4e0f-8283-c8e65365bf28

Table 14.1.1: Participant Disposition
All Participants

	SPVX02 (N = XX)		Tetadif (N = XX)		diTeBooster (N = XX)	
	n	(%)	n	(%)	n	(%)
Enrolled	XX		XX		XX	
Randomised	XX		XX		XX	
Safety Analysis Set [1]	XX	(XX.X)	XX	(XX.X)	XX	(XX.X)
Full Analysis Set [2]	XX	(XX.X)	XX	(XX.X)	XX	(XX.X)
Immunogenicity Analysis Set [3]	XX	(XX.X)	XX	(XX.X)	XX	(XX.X)
Completed Study [4]	XX	(XX.X)	XX	(XX.X)	XX	(XX.X)
Withdrew from Study	XX	(XX.X)	XX	(XX.X)	XX	(XX.X)
Reason for Withdrawal:						
Reason 1	XX	(XX.X)	XX	(XX.X)	XX	(XX.X)
…	XX	(XX.X)	XX	(XX.X)	XX	(XX.X)

[1] Safety Analysis Set = All randomised participants who were exposed to an investigational intervention.
[2] Full Analysis Set = All randomised participants.
[3] Immunogenicity Analysis Set = All randomised participants who were exposed to an investigational intervention and have at least one baseline and one corresponding post-baseline immunogenicity measurement and have no protocol deviations that may impact their immunogenicity results.
[4] Completed Study = Received study intervention and completed all study visits up to, and including, the follow-up visit.
Note: Percentages are based on the number of randomised participants.

Source: Listing 16.2.1.1 and 16.2.3.

Table 14.1.2: Demography
Safety Analysis Set

	Statistic	SPVX02 (N = XX)	Tetadif (N = XX)	diTeBooster (N = XX)
Sex				
Male	n (%)	XX (XX.X)	XX (XX.X)	XX (XX.X)
Female	n (%)	XX (XX.X)	XX (XX.X)	XX (XX.X)
Age at Informed Consent (years)	n	XX	XX	XX
	Mean (SD)	XX.X (XX.XX)	XX.X (XX.XX)	XX.X (XX.XX)
	Median	XX.X (XX.XX)	XX.X (XX.XX)	XX.X (XX.XX)
	Q1, Q3	XX.X	XX.X	XX.X
	Min, Max	XX.X	XX.X	XX.X
Ethnicity				
Hispanic or Latino	n (%)	XX (XX.X)	XX (XX.X)	XX (XX.X)
Not Hispanic or Latino	n (%)	XX (XX.X)	XX (XX.X)	XX (XX.X)
Not Reported	n (%)	XX (XX.X)	XX (XX.X)	XX (XX.X)
Unknown	n (%)	XX (XX.X)	XX (XX.X)	XX (XX.X)
Race				
Asian	n (%)	XX (XX.X)	XX (XX.X)	XX (XX.X)
American Indian or Alaska Native	n (%)	XX (XX.X)	XX (XX.X)	XX (XX.X)
Black or African American	n (%)	XX (XX.X)	XX (XX.X)	XX (XX.X)
Native Hawaiian or Other Pacific Islander	n (%)	XX (XX.X)	XX (XX.X)	XX (XX.X)
White	n (%)	XX (XX.X)	XX (XX.X)	XX (XX.X)
Other	n (%)	XX (XX.X)	XX (XX.X)	XX (XX.X)
Unknown	n (%)	XX (XX.X)	XX (XX.X)	XX (XX.X)
Child-Bearing Potential				
Yes	n (%)	XX (XX.X)	XX (XX.X)	XX (XX.X)
No	n (%)	XX (XX.X)	XX (XX.X)	XX (XX.X)
Height at Screening (cm)	n	XX	XX	XX
	Mean (SD)	XX.X (XX.XX)	XX.X (XX.XX)	XX.X (XX.XX)
	Median	XX.X (XX.XX)	XX.X (XX.XX)	XX.X (XX.XX)
	Q1, Q3	XX.X	XX.X	XX.X
	Min, Max	XX.X	XX.X	XX.X
[Repeat for, Weight at Screening (kg) and BMI at Screening (kg/m^2)]				

Note: Percentages are based on the number of non-missing observations.

Source: Listing 16.2.4.1.

E-Sign ID: dc6e1da8-9522-4e0f-8283-c8e65365bf28

Table 14.1.3: Medical History by System Organ Class and Preferred Term
Safety Analysis Set

System Organ Class<br>Preferred Term [1]	SPVX02<br>(N = XX)		Tetadif<br>(N = XX)		diTeBooster<br>(N = XX)	
	n	(%)	n	(%)	n	(%)
Number of Events	XX		XX		XX	
Number of Participants with any History	XX	(XX.X)	XX	(XX.X)	XX	(XX.X)
System Organ Class 1	XX	(XX.X)	XX	(XX.X)	XX	(XX.X)
Preferred Term 1	XX	(XX.X)	XX	(XX.X)	XX	(XX.X)
Preferred Term 2	XX	(XX.X)	XX	(XX.X)	XX	(XX.X)
System Organ Class 2	XX	(XX.X)	XX	(XX.X)	XX	(XX.X)
Preferred Term 1	XX	(XX.X)	XX	(XX.X)	XX	(XX.X)
Preferred Term 2	XX	(XX.X)	XX	(XX.X)	XX	(XX.X)

[1] MedDRA Version 28.0
Note: Percentages are based on the safety analysis set.

Source: Listing 16.2.4.3.

E-Sign ID: dc6e1da8-9522-4e0f-8283-c8e65365bf28

Table 14.1.4: Concomitant Medications
Safety Analysis Set

Drug Class (L2)/ WHO Drug Preferred Base Name [1]	SPVX02 (N = XX)		Tetadif (N = XX)		diTeBooster (N = XX)		Overall (N = XX)	
	n	(%)	n	(%)	n	(%)	n	(%)
Number of Participants with any Medication	XX	(XX.X)	XX	(XX.X)	XX	(XX.X)	XX	(XX.X)
Drug Class 1	XX	(XX.X)	XX	(XX.X)	XX	(XX.X)	XX	(XX.X)
WHO Drug Name 1	XX	(XX.X)	XX	(XX.X)	XX	(XX.X)	XX	(XX.X)
WHO Drug Name 2	XX	(XX.X)	XX	(XX.X)	XX	(XX.X)	XX	(XX.X)
Drug Class 2	XX	(XX.X)	XX	(XX.X)	XX	(XX.X)	XX	(XX.X)
WHO Drug Name 1	XX	(XX.X)	XX	(XX.X)	XX	(XX.X)	XX	(XX.X)
WHO Drug Name 2	XX	(XX.X)	XX	(XX.X)	XX	(XX.X)	XX	(XX.X)

[1] WHO Drug Dictionary Global version 2025 (March 2025). L2: The second level of Anatomical Therapeutic Chemical (ATC) classification.
Note: Concomitant medications are defined as those that were ongoing at the start of study intervention or started after the initiation of study intervention.
Note: Percentages are based on the safety analysis set.

Source: Listing 16.2.4.4.

Table 14.2.1.1.1: Seroprotection Rate - Diphtheria
Immunogenicity Analysis Set

	Statistic	SPVX02 (N = XX)	Tetadif (N = XX)	diTeBooster (N = XX)
Seroprotection	n (%)	XX (XX.X)	XX (XX.X)	XX (XX.X)
	95% CI	XX.X, XX.X	XX.X, XX.X	XX.X, XX.X
2-Fold Seroprotection	n (%)	XX (XX.X)	XX (XX.X)	XX (XX.X)
	95% CI	XX.X, XX.X	XX.X, XX.X	XX.X, XX.X
4-Fold Seroprotection	n (%)	XX (XX.X)	XX (XX.X)	XX (XX.X)
	95% CI	XX.X, XX.X	XX.X, XX.X	XX.X, XX.X

Note: Seroprotection is defined as IgG serum antibody titre of >= 0.1 IU/mL.
Note: 2-Fold seroprotection is defined as IgG serum antibody titre of >= 0.1 IU/mL and at least a 2-fold increase from baseline.
Note: 4-Fold seroprotection is defined as IgG serum antibody titre of >= 0.1 IU/mL and at least a 2-fold increase from baseline.
Note: Percentages are based on the immunogenicity analysis set.

Source: Listing 16.2.6.2.

This shell will be used for the following tables

Table 14.2.1.1.2: Seroprotection Rate - Diphtheria - Participants Not Seroprotected at Baseline
Table 14.2.1.1.3: Seroprotection Rate - Diphtheria - Participants Not Longer-Term Seroprotected at Baseline
Table 14.2.1.2.1: Seroprotection Rate - Tetanus
Table 14.2.1.2.2: Seroprotection Rate - Tetanus - Participants Not Seroprotected at Baseline
Table 14.2.1.2.3: Seroprotection Rate - Tetanus - Participants Not Longer-Term Seroprotected at Baseline

E-Sign ID: dc6e1da8-9522-4e0f-8283-c8e65365bf28

Table 14.2.2.1.1: Geometric Mean Antibodies - Diphtheria
Immunogenicity Analysis Set

	Statistic	SPVX02 (N = XX)	Tetadif (N = XX)	diTeBooster (N = XX)
Diphtheria Antibody Titre (IU/mL)				
Baseline	n	X	X	X
	Mean (SD)	XX.X (XX.XX)	XX.X (XX.XX)	XX.X (XX.XX)
	GM (GSD)	XX.X (XX.XX)	XX.X (XX.XX)	XX.X (XX.XX)
	Median	XX.X	XX.X	XX.X
	Q1, Q3	XX.X, XX.X	XX.X, XX.X	XX.X, XX.X
	Min, Max	XX, XX	XX, XX	XX, XX
Day 28	n	X	X	X
	Mean (SD)	XX.X (XX.XX)	XX.X (XX.XX)	XX.X (XX.XX)
	GM (GSD)	XX.X (XX.XX)	XX.X (XX.XX)	XX.X (XX.XX)
	Median	XX.X	XX.X	XX.X
	Q1, Q3	XX.X, XX.X	XX.X, XX.X	XX.X, XX.X
	Min, Max	XX, XX	XX, XX	XX, XX
Geometric Mean Ratio	GMR (GSD)	XX.X (XX.XX)	XX.X (XX.XX)	XX.X (XX.XX)
	95% CI	XX.X, XX.X	XX.X, XX.X	XX.X, XX.X

Source: Listing 16.2.6.1.

This shell will be used for

Table 14.2.2.1.2: Geometric Mean Antibodies - Diphtheria - By Seroprotection Status at Baseline
Table 14.2.2.1.3: Geometric Mean Antibodies - Diphtheria - By Longer-Term Seroprotection Status at Baseline
Table 14.2.2.1.4: Geometric Mean Antibodies - Diphtheria - By Age Group
Table 14.2.2.1.5: Geometric Mean Antibodies - Diphtheria - By Sex
Table 14.2.2.1.6: Geometric Mean Antibodies - Diphtheria - By BMI Group
Table 14.2.2.2.1: Geometric Mean Antibodies - Tetanus
Table 14.2.2.2.2: Geometric Mean Antibodies - Tetanus - By Seroprotection Status at Baseline
Table 14.2.2.2.3: Geometric Mean Antibodies - Tetanus - By Longer-Term Seroprotection Status at Baseline
Table 14.2.2.2.4: Geometric Mean Antibodies - Tetanus - By Age Group
Table 14.2.2.2.5: Geometric Mean Antibodies - Tetanus - By Sex
Table 14.2.2.2.6: Geometric Mean Antibodies - Tetanus - By BMI Group

Table 14.2.3.1.1: Longer-Term Seroprotection Rate - Diphtheria
Immunogenicity Analysis Set

	Statistic	SPVX02 (N = XX)	Tetadif (N = XX)	diTeBooster (N = XX)
Seroprotection	n (%)	XX (XX.X)	XX (XX.X)	XX (XX.X)
	95% CI	XX.X, XX.X	XX.X, XX.X	XX.X, XX.X
2-Fold Seroprotection	n (%)	XX (XX.X)	XX (XX.X)	XX (XX.X)
	95% CI	XX.X, XX.X	XX.X, XX.X	XX.X, XX.X
4-Fold Seroprotection	n (%)	XX (XX.X)	XX (XX.X)	XX (XX.X)
	95% CI	XX.X, XX.X	XX.X, XX.X	XX.X, XX.X

Note: Longer-term seroprotection is defined as IgG serum antibody titre of >= 1.0 IU/mL.
Note: 2-Fold longer-term seroprotection is defined as IgG serum antibody titre of >= 1.0 IU/mL and at least a 2-fold increase from baseline.
Note: 4-Fold longer-term seroprotection is defined as IgG serum antibody titre of >= 1.0 IU/mL and at least a 2-fold increase from baseline.
Note: Percentages are based on the immunogenicity analysis set.

Source: Listing 16.2.6.2.

This shell will be used for the following tables

Table 14.2.3.1.2: Longer-Term Seroprotection Rate - Diphtheria - Participants Not Seroprotected at Baseline
Table 14.2.3.1.3: Longer-Term Seroprotection Rate - Diphtheria - Participants Not Longer-Term Seroprotected at Baseline
Table 14.2.3.2.1: Longer-Term Seroprotection Rate - Tetanus
Table 14.2.3.2.2: Longer-Term Seroprotection Rate - Tetanus - Participants Not Seroprotected at Baseline
Table 14.2.3.2.3: Longer-Term Seroprotection Rate - Tetanus - Participants Not Longer-Term Seroprotected at Baseline

E-Sign ID: dc6e1da8-9522-4e0f-8283-c8e65365bf28

Table 14.3.1.1: Reactogenicity Events Summary
Safety Analysis Set

Symptom<br>Day	SPVX02<br>(N = XX)		Tetadif<br>(N = XX)		diTeBooster<br>(N = XX)		Overall<br>(N = XX)	
	n	(%)	n	(%)	n	(%)	n	(%)
Symptom 1								
Any	XX	(XX.X)	XX	(XX.X)	XX	(XX.X)	XX	(XX.X)
Day 1	XX	(XX.X)	XX	(XX.X)	XX	(XX.X)	XX	(XX.X)
Day 2	XX	(XX.X)	XX	(XX.X)	XX	(XX.X)	XX	(XX.X)
Day 3	XX	(XX.X)	XX	(XX.X)	XX	(XX.X)	XX	(XX.X)
Day 4	XX	(XX.X)	XX	(XX.X)	XX	(XX.X)	XX	(XX.X)
Day 5	XX	(XX.X)	XX	(XX.X)	XX	(XX.X)	XX	(XX.X)
Day 6	XX	(XX.X)	XX	(XX.X)	XX	(XX.X)	XX	(XX.X)
Day 7	XX	(XX.X)	XX	(XX.X)	XX	(XX.X)	XX	(XX.X)
[Repeat for each symptom]								
Any Symptom								
Any	XX	(XX.X)	XX	(XX.X)	XX	(XX.X)	XX	(XX.X)
Day 1	XX	(XX.X)	XX	(XX.X)	XX	(XX.X)	XX	(XX.X)
Day 2	XX	(XX.X)	XX	(XX.X)	XX	(XX.X)	XX	(XX.X)
Day 3	XX	(XX.X)	XX	(XX.X)	XX	(XX.X)	XX	(XX.X)
Day 4	XX	(XX.X)	XX	(XX.X)	XX	(XX.X)	XX	(XX.X)
Day 5	XX	(XX.X)	XX	(XX.X)	XX	(XX.X)	XX	(XX.X)
Day 6	XX	(XX.X)	XX	(XX.X)	XX	(XX.X)	XX	(XX.X)
Day 7	XX	(XX.X)	XX	(XX.X)	XX	(XX.X)	XX	(XX.X)

Note: Symptoms recorded by the participants in their eDiaries, summarised here, were interpreted by the Investigator and subsequently recorded as AEs.
Note: Percentages are based on the safety analysis set.

Source: Listing 16.2.7.1.

Table 14.3.1.2: Reactogenicity Events by Severity Summary
Safety Analysis Set

Symptom<br>Day	SPVX02<br>(N = XX)<br>n	 <br>(%)	Tetadif<br>(N = XX)<br>n	 <br>(%)	diTeBooster<br>(N = XX)<br>n	 <br>(%)	Overall<br>(N = XX)<br>n	 <br>(%)
Symptom 1								
Any								
Mild	XX	(XX.X)	XX	(XX.X)	XX	(XX.X)	XX	(XX.X)
Moderate	XX	(XX.X)	XX	(XX.X)	XX	(XX.X)	XX	(XX.X)
Severe	XX	(XX.X)	XX	(XX.X)	XX	(XX.X)	XX	(XX.X)
Day 1								
Mild	XX	(XX.X)	XX	(XX.X)	XX	(XX.X)	XX	(XX.X)
Moderate	XX	(XX.X)	XX	(XX.X)	XX	(XX.X)	XX	(XX.X)
Severe	XX	(XX.X)	XX	(XX.X)	XX	(XX.X)	XX	(XX.X)
Day 2								
Mild	XX	(XX.X)	XX	(XX.X)	XX	(XX.X)	XX	(XX.X)
Moderate	XX	(XX.X)	XX	(XX.X)	XX	(XX.X)	XX	(XX.X)
Severe	XX	(XX.X)	XX	(XX.X)	XX	(XX.X)	XX	(XX.X)
[Repeat for each day and for each symptom]								
Any Symptom								
Any								
Mild	XX	(XX.X)	XX	(XX.X)	XX	(XX.X)	XX	(XX.X)
Moderate	XX	(XX.X)	XX	(XX.X)	XX	(XX.X)	XX	(XX.X)
Severe	XX	(XX.X)	XX	(XX.X)	XX	(XX.X)	XX	(XX.X)
Day 1								
Mild	XX	(XX.X)	XX	(XX.X)	XX	(XX.X)	XX	(XX.X)
Moderate	XX	(XX.X)	XX	(XX.X)	XX	(XX.X)	XX	(XX.X)
Severe	XX	(XX.X)	XX	(XX.X)	XX	(XX.X)	XX	(XX.X)
Day 2								
Mild	XX	(XX.X)	XX	(XX.X)	XX	(XX.X)	XX	(XX.X)
Moderate	XX	(XX.X)	XX	(XX.X)	XX	(XX.X)	XX	(XX.X)
Severe	XX	(XX.X)	XX	(XX.X)	XX	(XX.X)	XX	(XX.X)
[Repeat for each day and for each symptom]								

Note: Symptoms recorded by the participants in their eDiaries, summarised here, were interpreted by the Investigator and subsequently recorded as AEs.
Note: For the Any Symptom summaries, if the participant experienced more than one symptom the worst severity will be summarised.
Note: Percentages are based on the safety analysis set.

Source: Listing 16.2.7.1.

Table 14.3.2.1: Overall Summary of Treatment-Emergent Adverse Events
Safety Analysis Set

	SPVX02 (N = XX)		Tetadif (N = XX)		diTeBooster (N = XX)		Overall (N = XX)	
Total Number of Participants	n	(%)	n	(%)	n	(%)	n	(%)
TEAEs	XX	(XX.X)	XX	(XX.X)	XX	(XX.X)	XX	(XX.X)
Related								
Related	XX	(XX.X)	XX	(XX.X)	XX	(XX.X)	XX	(XX.X)
Unrelated	XX	(XX.X)	XX	(XX.X)	XX	(XX.X)	XX	(XX.X)
Severity	XX	(XX.X)	XX	(XX.X)	XX	(XX.X)	XX	(XX.X)
Mild	XX	(XX.X)	XX	(XX.X)	XX	(XX.X)	XX	(XX.X)
Moderate	XX	(XX.X)	XX	(XX.X)	XX	(XX.X)	XX	(XX.X)
Severe	XX	(XX.X)	XX	(XX.X)	XX	(XX.X)	XX	(XX.X)
Serious TEAE	XX	(XX.X)	XX	(XX.X)	XX	(XX.X)	XX	(XX.X)
TEAE Leading to Discontinuation	XX	(XX.X)	XX	(XX.X)	XX	(XX.X)	XX	(XX.X)

Note: Treatment-Emergent Adverse Event (TEAE) is an AE occurring on or after the first dose of study intervention.
Note: If a participant experiences more than one event, then the event with the worst severity or strongest relationship is included in the relevant level of summarisation.
Note: Related AEs are those where the relationship has been recorded as possible, probable, unlikely, unknown or missing. Unrelated AEs are those recorded as not related.
Note: Adverse Event Leading to Discontinuation = Participant withdrew from the study due to an adverse event or serious adverse event.
Note: Percentages are based on the safety analysis set.

Source: Listing 16.2.7.2.

E-Sign ID: dc6e1da8-9522-4e0f-8283-c8e65365bf28

Table 14.3.2.2: Treatment-Emergent Adverse Events by System Organ Class and Preferred Term
Safety Analysis Set

System Organ Class<br>Preferred Term [1]	SPVX02 (N = XX)			Tetadif (N = XX)			diTeBooster (N = XX)			Overall (N = XX)		
	E	n	(%)	E	n	(%)	E	n	(%)	E	n	(%)
Number of Participants with any Adverse Event	XX	XX	(XX.X)	XX	XX	(XX.X)	XX	XX	(XX.X)	XX	XX	(XX.X)
System Organ Class 1	XX	XX	(XX.X)	XX	XX	(XX.X)	XX	XX	(XX.X)	XX	XX	(XX.X)
Preferred Term 1	XX	XX	(XX.X)	XX	XX	(XX.X)	XX	XX	(XX.X)	XX	XX	(XX.X)
Preferred Term 2	XX	XX	(XX.X)	XX	XX	(XX.X)	XX	XX	(XX.X)	XX	XX	(XX.X)
System Organ Class 2	XX	XX	(XX.X)	XX	XX	(XX.X)	XX	XX	(XX.X)	XX	XX	(XX.X)
Preferred Term 1	XX	XX	(XX.X)	XX	XX	(XX.X)	XX	XX	(XX.X)	XX	XX	(XX.X)
Preferred Term 2	XX	XX	(XX.X)	XX	XX	(XX.X)	XX	XX	(XX.X)	XX	XX	(XX.X)

[1] MedDRA Version 28.0
Note: Treatment-Emergent Adverse Event is an AE occurring on or after the first dose of study intervention.
Note: Table shows distinct number of participants with events (n) and number of events (E) for each system organ class/preferred term.
Note: Percentages are based on the safety analysis set.

Source: Listing 16.2.7.2.

E-Sign ID: dc6e1da8-9522-4e0f-8283-c8e65365bf28

Table 14.3.2.3: Treatment-Emergent Adverse Events by System Organ Class, Preferred Term and Severity
Safety Analysis Set

System Organ Class<br>Preferred Term [1]	SPVX02<br>(N = XX)			Tetadif<br>(N = XX)			diTeBooster<br>(N = XX)			Overall<br>(N = XX)		
	E	n	(%)	E	n	(%)	E	n	(%)	E	n	(%)
Number of Participants with any Adverse Event	XX	XX	(XX.X)	XX	XX	(XX.X)	XX	XX	(XX.X)	XX	XX	(XX.X)
System Organ Class 1												
Mild	XX	XX	(XX.X)	XX	XX	(XX.X)	XX	XX	(XX.X)	XX	XX	(XX.X)
Moderate	XX	XX	(XX.X)	XX	XX	(XX.X)	XX	XX	(XX.X)	XX	XX	(XX.X)
Severe	XX	XX	(XX.X)	XX	XX	(XX.X)	XX	XX	(XX.X)	XX	XX	(XX.X)
Preferred Term 1												
Mild	XX	XX	(XX.X)	XX	XX	(XX.X)	XX	XX	(XX.X)	XX	XX	(XX.X)
Moderate	XX	XX	(XX.X)	XX	XX	(XX.X)	XX	XX	(XX.X)	XX	XX	(XX.X)
Severe	XX	XX	(XX.X)	XX	XX	(XX.X)	XX	XX	(XX.X)	XX	XX	(XX.X)
Preferred Term 2												
Mild	XX	XX	(XX.X)	XX	XX	(XX.X)	XX	XX	(XX.X)	XX	XX	(XX.X)
Moderate	XX	XX	(XX.X)	XX	XX	(XX.X)	XX	XX	(XX.X)	XX	XX	(XX.X)
Severe	XX	XX	(XX.X)	XX	XX	(XX.X)	XX	XX	(XX.X)	XX	XX	(XX.X)

[1] MedDRA Version 28.0
Note: Treatment-Emergent Adverse Event is an AE occurring on or after the first dose of study intervention.
Note: Table shows distinct number of participants with events (n) and number of events (E) for each system organ class/preferred term/severity.
Note: If a participant experienced a specific event more than once then the event with the worst severity is summarised.
Note: Percentages are based on the safety analysis set.

Source: Listing 16.2.7.2.

This shell will be used for the following tables:

Table 14.3.2.5: Serious Treatment-Emergent Adverse Events by System Organ Class and Preferred Term
Table 14.3.2.6: Treatment-Emergent Adverse Events Leading to Death or Discontinuation by System Organ Class and Preferred Term

Table 14.3.2.4: Treatment-Emergent Adverse Events by System Organ Class, Preferred Term and Relationship to Study Intervention
Safety Analysis Set

System Organ Class<br>Preferred Term [1]	SPVX02<br>(N = XX)			Tetadif<br>(N = XX)			diTeBooster<br>(N = XX)			Overall<br>(N = XX)		
	E	n	(%)	E	n	(%)	E	n	(%)	E	n	(%)
Number of Participants with any Adverse Event	XX	XX	(XX.X)	XX	XX	(XX.X)	XX	XX	(XX.X)	XX	XX	(XX.X)
System Organ Class 1												
Related	XX	XX	(XX.X)	XX	XX	(XX.X)	XX	XX	(XX.X)	XX	XX	(XX.X)
Unrelated	XX	XX	(XX.X)	XX	XX	(XX.X)	XX	XX	(XX.X)	XX	XX	(XX.X)
Preferred Term 1												
Related	XX	XX	(XX.X)	XX	XX	(XX.X)	XX	XX	(XX.X)	XX	XX	(XX.X)
Unrelated	XX	XX	(XX.X)	XX	XX	(XX.X)	XX	XX	(XX.X)	XX	XX	(XX.X)
Preferred Term 2												
Related	XX	XX	(XX.X)	XX	XX	(XX.X)	XX	XX	(XX.X)	XX	XX	(XX.X)
Unrelated	XX	XX	(XX.X)	XX	XX	(XX.X)	XX	XX	(XX.X)	XX	XX	(XX.X)

[1] MedDRA Version 28.0
Note: Treatment-Emergent Adverse Event is an AE occurring on or after the first dose of study intervention.
Note: Table shows distinct number of participants with events (n) and number of events (E) for each system organ class/preferred term.
Note: Related AEs are those where the relationship has been recorded as possible, probable, unlikely, unknown or missing. Unrelated AEs are those recorded as not related.
Note: Percentages are based on the safety analysis set.

Source: Listing 16.2.7.2.

E-Sign ID: dc6e1da8-9522-4e0f-8283-c8e65365bf28

Table 14.3.3.1: Listing of Participants with Serious Adverse Events
Safety Analysis Set

Intervention / Participant Number / AE Number	MedDRA SOC / Preferred Term[1] / Adverse event (verbatim)	Start Date Time (Study day) / Stop Date Time (Study day)	TE [2]	Related [3] / Severity [4]
XXXX<br>XXXX<br>XX	XXXXXXXXX /<br>XXXXXX /<br>XXXXXX	DDMMMYYYY HH:MM (XX) /<br>DDMMMYYYY HH:MM (XX)	Yes or No	X /<br>X

[1] MedDRA Version 28.0.
[2] TE = Treatment-Emergent. An AE occurring on or after administration of study intervention.
[3] Related: 1 = Related; 2 = Probably Related; 3 = Possibly Related; 4 = Unlikely Related; 5 = Not Related.
[4] Severity: 1 = Mild; 2 = Moderate; 3 = Severe.

Source: Listing 16.2.7.2.

Table 14.3.3.2: Listing of Participants with Adverse Events Directly Resulting in Withdrawal
Safety Analysis Set

Treatment Sequence / Screening Number / AE Number	MedDRA SOC / Preferred Term [1] / Adverse event (verbatim)	Start Date Time (Study day) / Stop Date Time (Study day)	TE [2]	Related [3] / Severity [4]
XXXX<br>XXXX<br>XX	XXXXXXXXX /<br>XXXXXX /<br>XXXXXX	DDMMMYYYY HH:MM (XX) /<br>DDMMMYYYY HH:MM (XX)	Yes or No	X /<br>X

[1] MedDRA Version 28.0
[2] TE = Treatment-Emergent. An AE occurring on or after administration of study intervention.
[3] Related: 1 = Related; 2 = Probably Related; 3 = Possibly Related; 4 = Unlikely Related; 5 = Not Related.
[4] Severity: 1 = Mild; 2 = Moderate; 3 = Severe;

Source: Listing 16.2.7.2.

E-Sign ID: dc6e1da8-9522-4e0f-8283-c8e65365bf28

Table 14.3.4.1: Haematology – Change from Baseline Summary
Safety Analysis Set

		SPVX02 (N = XX)		Tetadif (N = XX)		diTeBooster (N = XX)	
Parameter (Units) Visit	Statistic	Absolute	Change from Baseline	Absolute	Change from Baseline	Absolute	Change from Baseline
XXXX (XXX)							
Baseline	n	X		X		X	
	Mean (SD)	XX.X (XX.XX)		XX.X (XX.XX)		XX.X (XX.XX)	
	Median	XX.X		XX.X		XX.X	
	Q1, Q3	XX.X, XX.X		XX.X, XX.X		XX.X, XX.X	
	Min, Max	XX, XX		XX, XX		XX, XX	
Follow-up	n	X	X	X	X	X	X
	Mean (SD)	XX.X (XX.XX)	XX.X (XX.XX)	XX.X (XX.XX)	XX.X (XX.XX)	XX.X (XX.XX)	XX.X (XX.XX)
	Median	XX.X	XX.X	XX.X	XX.X	XX.X	XX.X
	Q1, Q3	XX.X, XX.X	XX.X, XX.X	XX.X, XX.X	XX.X, XX.X	XX.X, XX.X	XX.X, XX.X
	Min, Max	XX, XX	XX, XX	XX, XX	XX, XX	XX, XX	XX, XX

Source: Listing 16.2.8.1.

This shell will be used for the following tables, with the source listing number updated as required:

Table 14.3.4.2: Chemistry – Change from Baseline Summary
Table 14.3.4.3: Coagulation – Change from Baseline Summary
Table 14.3.4.4: Thyroid Function – Change from Baseline Summary

Listing 16.2.1.1: Participant Disposition

Intervention	Participant Number/ Randomisation Number	Informed Consent Protocol Version/IC Version: Date/Time	Date of Randomisation	Date (Study Day) of Study Completion	Date (Study Day) of Early Withdrawal	Reason not Randomised/ Reason for Early Withdrawal
XXX	XXXXXX/ XXXXXX	X/X: DDMMMYYYY HH:MM	DDMMMYYYY	DDMMMYYYY (XX)	DDMMMYYYY (XX)	XXXXXXXXXXXXXXX

Programming Note: Include every re-consent

Programming Note: The last column should include any reasons for not randomizing or reasons for withdrawal.

Listing 16.2.1.2: Failed Inclusion and Exclusion Criteria

Participant Number	Reason for Exclusion	Comments
XXXXXX	XXXXXXXXXXXXXXXXXX	XXXXXXXXXXX

Programming note: Listing will state 'No Data to Report' if no participants fail the criteria.

E-Sign ID: dc6e1da8-9522-4e0f-8283-c8e65365bf28

Listing 16.2.2: Protocol Deviations

Intervention	Participant Number	Date Occurred (Study Day)	Deviation Description	Deviation Category	Other, Details
XXXXX	XXXXXX	DDMMMYYYY (XX)	XXXXXXXXX	XXXXXXX	XXXXXXXX

Listing 16.2.3: Participant Analysis Sets

Intervention	Participant Number	Safety Analysis Set [1]	Full Analysis Set [2]	Immunogenicity Analysis Set [3]
XXXXX	XXXXXX	Yes / No	Yes / No	Yes / No

[1] Safety Analysis Set = All randomised participants who were exposed to an investigational intervention.
[2] Full Analysis Set = All randomised participants.
[3] Immunogenicity Analysis Set = All randomised participants who were exposed to an investigational intervention and have at least one baseline and one corresponding post-baseline immunogenicity measurement and have no protocol deviations that may impact their immunogenicity results.

E-Sign ID: dc6e1da8-9522-4e0f-8283-c8e65365bf28

Listing 16.2.4.1: Demographic Characteristics and Body Measurements

Intervention	Participant Number	Age at informed consent (years)	Sex	Race / Ethnicity	Height (cm)	Weight (kg)	BMI (kg/m^2)
XXXXX	XXXXXX	xx	Male / Female	XXXXXX / XXXXXX	XXX	XX.X	XX.X

Programming Note: Height, Weight and BMI here are those collected at screening. The weight and BMI recorded at any other visits will be included in the vital signs listing.

E-Sign ID: dc6e1da8-9522-4e0f-8283-c8e65365bf28

Listing 16.2.4.2: Child-Bearing Potential and Contraception Use

Intervention	Participant Number	Child-Bearing Potential	If Not, Reason	Contraceptive Precautions	Other, Details
XXXXX	XXXXXX	Yes/No	XXXXXXXXX	XXXX / XXXX/ XXXX	XXXXXX

Note: ^ = Female.

Listing 16.2.4.3: Medical History

Intervention	Participant Number	MedDRA SOC / Preferred Term [1] / Medical Condition	Start Date/ End Date	Medical History ID
XXXXX	XXXXXX	XXXXXXXXX / XXXXXXXXX / XXXXXXXXX	DDMMMYYYY / DDMMMYYYY	XXX

[1] MedDRA Version 28.0.

Note: The modified term is the term that was used for MedDRA coding of the event where the event required multiple codes.

E-Sign ID: dc6e1da8-9522-4e0f-8283-c8e65365bf28

Listing 16.2.4.4: Prior and Concomitant Medications

Intervention	Participant Number	Drug Class (L2) / WHO Drug Preferred Base Name [1] / Medication (verbatim) <modified>	Start Date/Time (Study Day)/ End Date/Time (Study Day)	Indication / AE ID/ Med ID	Dose / Dose Units	Freq [2] / Route [3]	Prior or Concomitant[4]
XXXXX	XXXXXX	XXXXXXXXX / XXXXXXXXX / XXXXXXXXXXXX	DDMMMYYYY HH:MM (XX) / DDMMMYYYY HH:MM (XX)	XXXXXX / AEn / MHn	XXX / XXX	XXX / XXX	P / C

[1] WHO Drug Dictionary Global version 2025 (March 2025). L2: The second level of Anatomical Therapeutic Chemical (ATC) classification.
[2] OD = Once a day, BD = Twice a day, PRN = As needed, TDS = Three times a day, QDS = Four times a day, CTS = Continuous.
[3] A = Aural, IM = Intramuscular, INH = Inhalation, IUD = Intrauterine Device, IV = Intravenous, N = Nasal, O = Ocular, PO = Oral, PR = Rectal, PV = Vaginal, SC = Subcutaneous, SL = Sublingual, TOP = Topical.
[4] If the medication stop date is before the date of study intervention the medication will be assigned as being prior (P). In all other cases, the medication is assigned as being concomitant with study intervention (C).

Programming Note: Please update the footnotes and codes for [2] and [3] to match what is seen in the data.

E-Sign ID: dc6e1da8-9522-4e0f-8283-c8e65365bf28

Listing 16.2.4.5: Substance Use History

Intervention	Participant Number	Smoking History	Alcohol History
XXXXX	XXXXXX	Never/Past/Current	Past/Current

Listing 16.2.5.1: Study Drug Administration

Intervention	Participant Number	Administered (as Per Protocol)?	Reason not Administered (as Per Protocol)	Administration Date/Time (Study Day)/	Location of Administration	Monitored for Required Time?	If Not Monitored, Reason
XXXXX	XXXXXX	Yes/No	XXXXXXXXXXXXX	DDMMMYYYY HH:MM (XX)	Left Arm/<br>Right Arm	Yes/No	XXXXXXXXXXXXX

Note: Required observation time is at least 1 hour for participants in the sentinel group and at least 30 mins for all other participants.

E-Sign ID: dc6e1da8-9522-4e0f-8283-c8e65365bf28

Listing 16.2.5.2: Post-Administration Observation

Intervention	Participant Number	Symptoms	Experienced?	Severity	Measurement[1]
XXXXX	XXXXXX	XXXXXXXX	Yes/No	Mild/Moderate/Severe	XX.X

[1] Measurement is only expected for temperature (Degrees C), induration/swelling (cm) and erythema/redness (cm).

E-Sign ID: dc6e1da8-9522-4e0f-8283-c8e65365bf28

Listing 16.2.6.1: Antibody Titres

Intervention	Participant Number	Disease	Visit	Assessment Date/Time (Study Day)	Titre	Change from Baseline	Ratio of Baseline	Sero-Protected at Baseline	Longer-Term Sero-Protected at Baseline	Comments
XXXXX	XXXXXX	Tetanus/ Diphtheria	Day 1	DDMMMYYYY HH:MM (XX)	XX.XX			Yes/No	Yes/No	
XXXXX	XXXXXX		Day 28	DDMMMYYYY HH:MM (XX)	XX.XX	XX.XX	XX.XX			

Note: # = Baseline.

Programming Note: Comments column should include any comments from external data or reasons not done.
Programming Note: Seroprotection at baseline should appear on the baseline row for each of diphtheria and tetanus

E-Sign ID: dc6e1da8-9522-4e0f-8283-c8e65365bf28

Listing 16.2.6.2: Seroprotection

Intervention	Participant Number	Disease	Seroprotection	Long-Term Seroprotection	2-Fold Seroprotection	2-Fold Long-Term Seroprotection	4-Fold Seroprotection	4-Fold Long-Term Seroprotection
XXXXX	XXXXXX	Tetanus/Diphtheria	Yes/No	Yes/No	Yes/No	Yes/No	Yes/No	Yes/No

E-Sign ID: dc6e1da8-9522-4e0f-8283-c8e65365bf28

Listing 16.2.7.1: Reactogenicity Events

Intervention	Participant Number	e-Diary Day	Date/Time Completed	Temp [1]	Symptoms	Experienced ?	Interference with Activities	Measurement [2]	Medication?	A&E/Hospital?	Severity Grade
XXXXX	XXXXXX	Day X	DDMMMYYYY HH:MM		XXXXXXXX	Yes/No	None/Some/Significant	XXX	Yes/No	Yes/No	Mild/ Moderate/ Severe

[1] Temperature is measured at the point of diary entry. Fever severity is defined as: Mild = 38.0-38.4; Moderate = 38.5-38.9; Severe = 39.0-40.0; Potentially Life Threatening = >40.0.
[2] Measurement is only expected for feverishness (Degrees C), induration/swelling (cm) and erythema/redness (cm), where it is reported that the symptom is experienced.

Listing 16.2.7.2: Adverse Events

Intervention / Participant Number / AE Number	MedDRA SOC / Preferred Term[1] / Adverse Event (verbatim)	Start Date/Time (Study Day) / Stop Date/Time (Study Day)	TE [2]	Related [3] / Severity [4]	SAE[5] / Serious Criteria[6]	Outcome[7] / Action Taken [8]/ Other Actions, Details
XXXX<br>XXXX<br>XX	XXXXXXXXX /<br>XXXXXX /<br>XXXXXX	DDMMMYYYY HH:MM (XX) /<br>DDMMMYYYY HH:MM (XX)	Yes or No	X /<br>X	Yes or No /<br>XX	X /<br>X /<br>XXXXXXXXXXXXXXXXXX

[1] MedDRA Version 28.0.
[2] TE = Treatment-Emergent. An AE occurring on or after administration of study intervention.
[3] Related: 1 = Definitely Related; 2 = Probably Related; 3 = Possibly Related; 4 = Unlikely Related; 5 = Not Related.
[4] Severity: 1 = Mild; 2 = Moderate; 3 = Severe;
[5] SAE = Serious Adverse Event.
[6] Serious Criteria: 1 = Death; 2 = Life-threatening; 3 = Hospitalization; 4 = Persistent or significant disability/incapacity; 5 = Congenital anomaly/birth defect; 6 = Other.
[7] Outcome: 1 = Recovered/Resolved; 2 = Recovered/Resolved with Sequelae; 3 = Recovering/Resolving; 4 = Not Recovered/Not Resolved; 5 = Fatal; 6 = Unknown.
[8] Action Taken: 1 = None; 2 = Non-drug therapy given; 3 = Concomitant medication taken; 4 = Participant withdrawn; 5 = Participant hospitalised; 6 = Other

Programming Note: Update the footnotes to match what is in the final data

E-Sign ID: dc6e1da8-9522-4e0f-8283-c8e65365bf28

Listing 16.2.8.1: Haematology

Intervention	Participant Number	Visit	Sample Collection Date/Time (Study Day)	Parameter	Result	Change from Baseline	Units	Lower Limit	Upper Limit	Interpretation	Comments
XXXXX	XXXXXX	XXX	DDMMMYYYY HH:MM (XX)	Basophils<br>XXXX<br>XXXX<br>XXXX	X.XX	X.XX	X10^9/L	X.XX	X.XX	Normal/<br>Abnormal NCS /<br>Abnormal CS	XXXXXXXXXXXX

Note: # = Baseline; CS = Clinically Significant; NCS = Not Clinically Significant.
Note: Values prefixed with < are treated as 0 when calculating the change from baseline.

Programming Note: The parameters should be listed in alphabetical order.
Programming Note: # should be placed against the baseline measurement for each parameter.
Programming Note: Comments column should include any comments from external data or reasons not done.

The same listing template will be used for

Listing 16.2.8.2: Chemistry
Listing 16.2.8.3: Coagulation
Listing 16.2.8.4: Thyroid Function

E-Sign ID: dc6e1da8-9522-4e0f-8283-c8e65365bf28

Listing 16.2.8.5: Urinalysis

Intervention	Participant Number	Visit	Sample Collection Date/Time (Study Day)	Parameter	Result	Comments
XXXXX	XXXXXX	XXX	DDMMMYYYY HH:MM (XX)	Basophils	X.XX	XXXXXXXXXXXX
				XXXX		
				XXXX		
				XXXX		

Programming Note: The parameters should be listed in alphabetical order.

E-Sign ID: dc6e1da8-9522-4e0f-8283-c8e65365bf28

Listing 16.2.9.1: Pregnancy Tests

Intervention	Participant Number	Visit	Test Date/Time (Study Day)	Sample Type	Result
XXXXX	XXXXXX	XXX	DDMMMYYYY HH:MM (XX)	Urine/Serum	Positive / Negative

E-Sign ID: dc6e1da8-9522-4e0f-8283-c8e65365bf28

Listing 16.2.9.2: Drugs of Abuse Screen

Intervention	Participant Number	Visit	Sample Collection Date/Time (Study Day)	Drug	Result	Reason Not Done
XXXXX	XXXXXX	XXX	DDMMMYYYY HH:MM (XX)	XXXXX	Positive / Negative / Not done	XXXXXXXX

Listing 16.2.9.3: Viral Serology

Intervention	Participant Number	Visit	Sample Collection Date/Time (Study Day)	Parameter	Result	Reason not Done
XXXXX	XXXXXX	XXX	DDMMMYYYY HH:MM (XX)	HIV	Positive / Negative	XXXXXXXX
				HBsAg	Positive / Negative	XXXXXXXX
				HCV	Positive / Negative	XXXXXXXX

E-Sign ID: dc6e1da8-9522-4e0f-8283-c8e65365bf28

Listing 16.2.10: Vital Signs

Intervention	Participant Number	Visit	Date/Time (Study Day)	Parameter	Method	Result	Units	Reason Not Done
XXXXX	XXXXXX	XXX	DDMMMYYYY HH:MM (XX)	XXXX	Tympani/Oral	XX	XX	XXXXXXXXX
				XXXX		XX	XX	
				XXXX		XX	XX	
				XXXX		XX	XX	

Programming Note: Method is only relevant to temperature

E-Sign ID: dc6e1da8-9522-4e0f-8283-c8e65365bf28

Listing 16.2.11: Electrocardiogram

Intervention	Participant Number	Visit	Date/Time (Study Day)	Parameter	Result	Units	Interpretation Detail	Reason Not Done
XXXXX	XXXXXX	XXX	DDMMMYYYY / HH:MM (XX)	XXX<br>XXX<br>XXX<br>Interpretation [1]	XXX	XXXX	XXXXXXXXX	XXXXXXXXX

[1] NCS = Not clinically significant, CS = Clinically significant.

Programming Note: The interpretation will be shown as an additional parameter, with the interpretation outcome shown in the result column.

Listing 16.2.12: Physical Examination

Intervention	Participant Number	Visit	Assessment Date/Time (Study Day)	System	Interpretation [1]	Interpretation Detail	Reason not Done
XXXXX	XXXXXX	XXX	DDMMMYYYY / HH:MM (XX)	XXX	XXX	XXXXXXXXX	XXXXXXXXX

[1] NCS = Not clinically significant, CS = Clinically significant.

E-Sign ID: dc6e1da8-9522-4e0f-8283-c8e65365bf28

Listing 16.2.13: Visit Dates

Intervention	Participant Number	Visit	Date	Source of Unscheduled Visit	Comments
XXXXX	XXXXXX	XXX	DDMMMYYYY	XXXXXXXXX	XXXXXXXXXXXX

Programming note: Unscheduled visits and visit information will be taken from specific database modules and the "Reason for additional collection" will be placed in the comments column in this listing.

This Document has been Signed with a **secure electronic signature** via E-Sign.

Certification Of Completion

**Title**	SPL-SPVX02-01 - Final Statistical Analysis Plan
**Author**	Laura Grey (lag@s-cubed.co.uk)
**Envelope Created on**	Fri, 12 Sep 2025 15:14:43
**Envelope ID**	dc6e1da8-9522-4e0f-8283-c8e65365bf28

Document Details

**Title**	SPL-SPVX02-01_Stablepharma_SAP_Final_1.00_12Sep2025.pdf.pdf
**Digital Fingerprint**	7a4a87e5-25e6-4c0c-bba4-b6f5e8b16a67

Document Signers Scan/Click the QR Code to view signature information

**Name**	Laura Grey
**Email**	lag@s-cubed.co.uk
**Status**	**SIGNED** at Fri, 12 Sep 2025 15:14:56 BST(+0100)
**Signature Fingerprint**	9d93d39b-8d10-4db9-a875-18ef874b950d
**Signing Reason**	I am the owner of this document

**Name**	Karen O'Hanlon
**Email**	kohanlon@stablepharma.com
**Status**	**SIGNED** at Tue, 16 Sep 2025 10:06:26 BST(+0100)
**Email signer verification**	**kohanlon@stablepharma.com**
**Signature Fingerprint**	b16b2fe9-43b2-4c6e-b9e2-4b32efd99686
**Signing Reason**	I approve this document

**Name**	Juana De La Torre Arrieta
**Email**	jtorre@stablepharma.com
**Status**	**SIGNED** at Mon, 15 Sep 2025 09:09:41 BST(+0100)
**Email signer verification**	**jtorre@stablepharma.com**
**Signature Fingerprint**	3e51565f-d7bd-49c6-9f3e-f17090b53f05
**Signing Reason**	I approve this document

Signer Events

**Tue, 16 Sep 2025 10:06:27**	**Karen O'Hanlon** Signed the Document (IP: 90.222.114.193)
**Mon, 15 Sep 2025 09:09:41**	**Juana De La Torre Arrieta** Signed the Document (IP: 84.121.249.69)
**Fri, 12 Sep 2025 15:14:56**	Secured by document PIN
**Fri, 12 Sep 2025 15:14:56**	**Laura Grey** Signed the Document (IP: 86.144.147.214)

British Assessment Bureau
UKAS MANAGEMENT SYSTEMS 8289
ISO 27001
INFORMATION SECURITY MANAGEMENT
